# Protein Interactomes of Streptococcus mutans YidC1 and YidC2 Membrane Protein Insertases Suggest SRP Pathway-Independent- and -Dependent Functions, Respectively

**DOI:** 10.1128/mSphere.01308-20

**Published:** 2021-03-03

**Authors:** Patricia Lara Vasquez, Surabhi Mishra, Senthil K. Kuppuswamy, Paula J. Crowley, L. Jeannine Brady

**Affiliations:** a Department of Oral Biology, University of Florida, Gainesville, Florida, USA; University of Wisconsin—Madison

**Keywords:** *Streptococcus mutans*, YidC, interactomes, membrane proteins, protein translocation, signal recognition particle

## Abstract

Virulence properties of cariogenic Streptococcus mutans depend on integral membrane proteins. Bacterial cotranslational protein trafficking involves the signal recognition particle (SRP) pathway components Ffh and FtsY, the SecYEG translocon, and YidC chaperone/insertases. Unlike Escherichia coli, S. mutans survives loss of the SRP pathway and has two *yidC* paralogs. This study characterized YidC1 and YidC2 interactomes to clarify respective functions alone and in concert with the SRP and/or Sec translocon. Western blots of formaldehyde cross-linked or untreated S. mutans lysates were reacted with anti-Ffh, anti-FtsY, anti-YidC1, or anti-YidC2 antibodies followed by mass spectrometry (MS) analysis of gel-shifted bands. Cross-linked lysates of wild-type and Δ*yidC2* strains were reacted with anti-YidC2-coupled Dynabeads, and cocaptured proteins were identified by MS. Last, YidC1 and YidC2 C-terminal tail-captured proteins were subjected to two-dimensional (2D) difference gel electrophoresis and MS analysis. Direct interactions of putative YidC1 and YidC2 binding partners were confirmed by bacterial two-hybrid assay. Our results suggest YidC2 works preferentially with the SRP pathway, while YidC1 is preferred for SRP-independent Sec translocon-mediated translocation. YidC1 and YidC2 autonomous pathways were also apparent. Two-hybrid assay identified interactions between holotranslocon components SecYEG/YajC and YidC1. Both YidC1 and YidC2 interacted with Ffh, FtsY, and chaperones DnaK and RopA. Putative membrane-localized substrates HlyX, LemA, and SMU_591c interacted with both YidC1 and YidC2. Identification of several Rgp proteins in the YidC1 interactome suggested its involvement in bacitracin resistance, which was decreased in Δ*yidC1* and SRP-deficient mutants. Collectively, YidC1 and YidC2 interactome analyses has further distinguished these paralogs in the Gram-positive bacterium S. mutans.

**IMPORTANCE** Streptococcus mutans is a prevalent oral pathogen and major causative agent of tooth decay. Many proteins that enable this bacterium to thrive in its environmental niche and cause disease are embedded in its cytoplasmic membrane. The machinery that transports proteins into bacterial membranes differs between Gram-negative and Gram-positive organisms, an important difference being the presence of multiple YidC paralogs in Gram-positive bacteria. Characterization of a protein’s interactome can help define its physiological role. Herein, we characterized the interactomes of S. mutans YidC1 and YidC2. Results demonstrated substantial overlap between their interactomes but also revealed several differences in their direct protein binding partners. Membrane transport machinery components were identified in the context of a large network of proteins involved in replication, transcription, translation, and cell division/cell shape. This information contributes to our understanding of protein transport in Gram-positive bacteria in general and informs our understanding of S. mutans pathogenesis.

## INTRODUCTION

Dental caries is the most common infectious disease in the world (1). Tooth decay occurs when acidogenic bacteria on the tooth surface take up and ferment dietary sugars, producing organic acids that cause enamel demineralization. A major agent of caries, Streptococcus mutans, is acidogenic and aciduric, enabling this species to tolerate acid and outcompete other oral microbes. S. mutans displays inherent characteristics that promote dominance in its ecological niche, including efficient carbohydrate uptake and fermentation, sucrose-dependent and sucrose-independent adhesins leading to biofilm formation, robust acid tolerance mechanisms, and quorum-sensing systems involved in bacteriocin production and genetic competence (2). These processes depend on integral membrane proteins and/or membrane-associated proteins. S. mutans’ competitive advantage and virulence attributes stem from its ability to sense and adapt to the harsh conditions it faces in the oral cavity. Efficient protein transport into and through the membrane is an essential aspect of this adaptability.

In bacteria, many integral membrane proteins are inserted into the cytoplasmic membrane cotranslationally using the signal recognition particle (SRP) pathway conserved in all living cells (reviewed in reference 3). The SRP binds hydrophobic signal sequences of nascent polypeptide substrates as they emerge from the ribosome. The bacterial ribosome-nascent chain (RNC) complex is targeted to the membrane via a transient interaction of the SRP protein, Ffh, with the bacterial SRP receptor, FtsY. This docks the RNC with the SecYEG translocon pore and enables translocation of the substrate into the membrane concomitant with translation. In addition to SecYEG, the integral membrane protein YidC also participates in membrane protein integration (4). YidC belongs to the Oxa/Alb/YidC family of insertases found in mitochondria, chloroplasts, and bacteria. Membrane biogenesis has been most widely studied in the Gram-negative bacterium Escherichia coli; however, studies of Gram-positive bacteria such as S. mutans and *Bacillus* spp. have revealed differences in the translocation machineries of Gram-negative and Gram-positive organisms (5). Importantly, Gram-positive bacteria almost universally encode two, or occasionally more, YidC paralogs. Gram-negative organisms possess a single YidC.

The SRP pathway is dispensable in S. mutans, although its disruption results in growth impairment, environmental stress sensitivity, and diminished genetic competence (6, 7). Deletion of S. mutans *yidC2* causes a similar phenotype, whereas deletion of *yidC1* appears less detrimental (7–10). YidC is essential in E. coli (11). S. mutans survives elimination of *yidC1* or *yidC2*, but a double mutant is not viable. A double *yidC2 ffh* deletion mutant is also not viable. In contrast, an *ffh yidC1* mutant is viable, albeit severely stress sensitive and growth impaired (10). These results suggest synthetic lethality and functional redundancies between the SRP pathway and YidC2 and between YidC1 and YidC2. While YidC1 and YidC2 apparently substitute for one another in some cases, distinct functional activities have been identified. S. mutans YidC1 impacts cell surface biogenesis and bacterial adhesion more than YidC2, while YidC2 impacts cell wall biosynthesis and localization of penicillin-binding proteins to the division septum (9, 10). Other Gram-positive organisms also demonstrate functional differences associated with multiple YidC paralogs. Phenotypic characterization of dual YidCs in Bacillus subtilis showed the significance of YidC1 in sporulation and YidC2 in competence (12, 13). Available sequence and structural data of Gram-positive bacterial YidCs do not totally explain their differences in function, although appending the longer and more positively charged C-terminal tail of YidC2 onto YidC1 can partially alleviate the S. mutans Δ*yidC2* stress-sensitive phenotype (8).

Distinct and overlapping functions of each YidC paralog in the physiology of S. mutans, and Gram-positive bacteria in general, can be revealed by identifying their respective interaction partners. Such a study can lend insight not only on specific clients of YidC1 and YidC2 as related to bacterial physiology but can also help in more fully characterize their roles in separate protein translocation pathways. In E. coli, its sole YidC can work independently (11, 14, 15), in collaboration with the Sec machinery (16–18), and in collaboration with SRP pathway components (18, 19). E. coli membrane proteins inserted by YidC alone are relatively few and generally contain only one or two transmembrane (TM) domains (20–25). Insertion of larger membrane proteins requires the Sec machinery and YidC (19, 26). The respective substrates of integrated YidC/SRP, SecYEG/SRP, and YidC/SecYEG/SRP pathways are largely unknown. Comparison of the membrane proteomes of S. mutans wild-type and mutant strains lacking *ffh*, *yidC1*, *yidC2*, or *ffh yidC1* suggested that its SRP pathway works in concert with YidC2 or YidC1 specifically, or with no preference, to insert most membrane-localized substrates (10). In a few instances, only the SRP pathway, or only YidC1 or YidC2, appeared to be required (10). In the current study, we began with an unbiased screening approach to evaluate similarities and differences between protein interactomes of S. mutans YidC1 and YidC2 within whole-cell lysates. This led to identification of potential substrates and revealed interaction networks, including proteins associated with translation as well as transcription and DNA replication. Bacterial two-hybrid assays to evaluate direct protein binding to YidC1 and YidC2 demonstrated interactions of both insertases with putative substrates ComA, Smu_591c, HlyX, and LemA. Two-hybrid experiments with chaperones and holotranslocon components also revealed overlap in their ability to partner with YidC1and YidC2, with the exceptions of SecY, SecE, and YajC that interacted exclusively with YidC1. Collectively, results of the current study, particularly in the context of previous genetic and membrane proteomic studies (7, 8, 10), indicate that YidC2 functions primarily in concert with the SRP pathway, while YidC1 is the preferred SecYEG partner in the absence of the SRP.

## RESULTS AND DISCUSSION

### Identification of potential binding partners of S. mutans YidC1, YidC2, Ffh, and/or FtsY in whole-cell lysates by formaldehyde cross-linking and Western blot gel shift.

**FIG 1:**
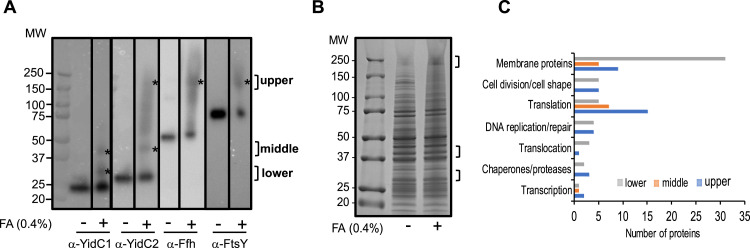
Formaldehyde cross-linking of S. mutans results in gel shifts of protein translocation machinery components present in whole-cell lysates. (A) Whole-cell lysates of untreated (−) S. mutans strain UA159 or cells treated with 0.4% formaldehyde (FA) (+) were analyzed by Western blotting using anti-YidC1 (α-YidC1), anti-YidC2, anti-Ffh, and anti-FtsY antibodies. Brackets and asterisks indicate regions of reactivity subjected to further analysis. Bands corresponding to YidC1 (24 kDa), YidC2 (27 kDa), Ffh (54 kDa), and FtsY (75 kDa) are apparent in untreated and formaldehyde cross-linked samples. MW, molecular weight. (B) Corresponding Coomassie blue-stained SDS-polyacrylamide gel indicating location of excised gel slices sent for mass spectrometry analysis. (C) Histogram showing number of proteins in indicated categories in upper, middle, and lower excised gel slices.

As a first step toward identifying putative binding partners and/or substrates of YidC1, YidC2, and the SRP pathway, we utilized the cell-penetrating cross-linking agent formaldehyde. After cross-linking, whole-cell lysates were prepared from S. mutans strain UA159, separated by sodium dodecyl sulfate-polyacrylamide gel electrophoresis (SDS-PAGE), and potential regions of interest were identified by Western blotting with anti-YidC1, anti-YidC2, anti-Ffh, and anti-FtsY antibodies (Fig. 1A). Bands corresponding to YidC1, YidC2, Ffh, and FtsY were readily identified in both cross-linked and non-cross-linked samples. Western blotting also revealed several regions of gel-shifted antibody reactivity in the formaldehyde cross-linked sample compared to the non-cross-linked sample (Fig.1A). Three distinct gel-shifted regions were excised from corresponding Coomassie blue-stained SDS-polyacrylamide gels for mass spectrometric (MS) analysis (Fig. 1B). These regions included a high-molecular-weight region (∼200 to 250 kDa) reactive with anti-YidC2, anti-Ffh, and anti-FtsY antibodies, but not anti-YidC1 antibodies, a middle region of ∼40 to 45 kDa reactive with both anti-YidC1 and anti-YidC2 antibodies, and a lower region of ∼30 to 33 kDa reactive only with anti-YidC1 antibody (Fig. 1A). Proteins present in the upper, middle, and lower molecular weight (MW) gel slices of the cross-linked sample, but not detected in the corresponding non-cross-linked control sample, are summarized in Table S1 in the supplemental material. Initially, MS analysis was performed only on the upper and middle molecular weight gel-shifted regions, but because relatively few proteins were identified in that experiment, we moved on to the immunocapture approach described below to improve sensitivity. During that time period, a more sensitive mass spectrometer became available, and the experiment was repeated with analysis of all three molecular weight regions. The results presented include the combined data from both experiments.

10.1128/mSphere.01308-20.5 TABLE S1 S. mutans proteins present in gel-shifted bands identified by Western blotting of formaldehyde-cross-linked but not non-cross-linked whole-cell lysates reacted with anti-YidC1, anti-YidC2, anti-Ffh, and anti-FtsY antibodies. Download Table S1, PDF file, 0.4 MB. Copyright © 2021 Vasquez et al. 2021 Vasquez et al. https://creativecommons.org/licenses/by/4.0/ This content is distributed under the terms of the Creative Commons Attribution 4.0 International license.

A total of 65, 38, and 119 proteins were identified in the upper, middle, and lower molecular weight gel slices, respectively, of the cross-linked but not non-cross-linked samples (Table S1). The lower region, recognized only by anti-YidC1 antibodies, contained the highest proportion of membrane proteins (31/119). These proteins may therefore represent substrates of a pathway that involves YidC1, but not YidC2, during growth under non-stress conditions. In contrast, the higher molecular weight region recognized by anti-Ffh, anti-FtsY, and anti YidC2 antibodies, and the middle region recognized by anti-YidC1 and anti-YidC2 antibodies, contained fewer membrane proteins, 9/65 and 5/38, respectively. Most of the non-integral membrane proteins identified in all three molecular weight regions were previously shown to be membrane associated during proteomic analysis of membranes derived from wild-type S. mutans and Δ*yidC1*, Δ*yidC2*, Δ*ffh*, and Δ*ffh yidC1* mutant strains (10). Many membrane-associated proteins are components of multimeric membrane-localized complexes that also contain integral membrane components. Thus, identification of membrane-associated proteins may therefore indirectly reflect the actual integral membrane protein substrates. We also identified multiple proteins involved in DNA replication and repair, transcription, translation, and cell division suggesting an extensive coordinated cellular machinery that includes membrane protein translocation (Table 1). Figure 1C depicts the distribution of proteins identified in each of the three molecular weight regions into functional categories.

**TABLE 1:** Summary of proteins present in upper, middle, and lower molecular weight regions, identified by Western blot gel shift assays of whole-cell lysates of formaldehyde-cross-linked S. mutans detected with anti-YidC1, anti-YidC2, anti-Ffh, and anti-FtsY-specific antibodies^*a*^

Category	Upper region (reactive with anti-YidC2, anti-Ffh, and anti-FtsY antibodies)	Middle region (reactive with YidC1 and YidC2)	Lower region (reactive with anti-YidC1 antibodies)
Translation	50S ribosomal protein L1 (SMU_1626)*	50S ribosomal protein L1 (SMU_1626)*	Putative ribosomal protein S1; sequence-specific DNA-binding protein (SMU_1200)§
	50S ribosomal protein L2 (SMU_2167)*	50S ribosomal protein L2 (SMU_2167)*	Dimethyladenosine transferase, 16S rRNA methyltransferase (SMU_349)
	50S ribosomal protein L7/L12 (SMU_960)	50S ribosomal protein L5 (SMU_2015)	Putative tRNA pseudouridine synthase A (SMU_84)
	Putative ribosomal protein S1; sequence-specific DNA-binding protein (SMU_1200)§	50S ribosomal protein L18 (SMU_2010)	Translation elongation factor EF-Tu (SMU_714)§
	30S ribosomal protein S3 (SMU_2021)*	30S ribosomal protein S2 (SMU_2032)	Conserved hypothetical protein, 16S rRNA methyltransferase (SMU_1659c)
	30S ribosomal protein S5 (SMU_2009)	30S ribosomal protein S3 (SMU_2021)*	
	30S ribosomal protein S10 (SMU_2026c)	30S ribosomal protein S13 (SMU_2003)	
	30S ribosomal protein S11 (SMU_2002)		
	30S ribosomal protein S18 (SMU_1858)		
	Translation elongation factor EF-Tu (SMU_714)§		
	Translation elongation factor G (SMU_359)		
	Putative translation elongation factor TS (SMU_2031)		
	Translation initiation factor 2 (SMU_421)		
	Putative translation initiation factor IF3 (SMU_697)		
	Putative alanyl-tRNA synthetase (alanine-tRNA ligase) (SMU_650)		

Translocation	Preprotein translocase subunit SecA (SMU_1838)		Putative preprotein translocase SecY protein (SMU_2006)
			Putative secreted protein, preprotein translocase subunit YajC (SMU_1787c)
			Putative signal peptidase II (SMU_1661c)

Chaperones/proteases	Peptidyl-prolyl isomerase RopA (trigger factor) (SMU_91)§		Peptidyl-prolyl isomerase RopA (trigger factor) (SMU_91)§
	Heat shock protein. DnaK (HSP-70) (SMU_82)		Putative chaperonin GroEL (SMU_1954)§
	Putative chaperonin GroEL (SMU_1954)§		

Cell division/cell shape	Putative septation ring formation regulator (SMU_1276c)		Putative cell shape-determining protein MreC (SMU_20)
	Putative cell division protein DivIVA (SMU_557)		Putative cell division protein RodA (SMU_1279c)
	Putative cell division protein FtsZ (SMU_552)		Integral membrane protein possibly involved in d-alanine export, d-alanyl-lipoteichoic acid biosynthesis protein DltB (SMU_1690)
	Cell division protein FtsA (SMU_551)		Putative cell division protein FtsH (SMU_15)§
	Putative cell division protein FtsH (SMU_15)§		Conserved hypothetical protein cell division protein FtsW (SMU_172)

DNA replication/repair	DNA-directed RNA polymerase, alpha subunit (SMU_2001)		Putative type II restriction endonuclease (SMU_506)
	DNA-dependent RNA polymerase, beta subunit (SMU_1990)		Putative site-specific DNA methyltransferase (SMU_504)
	DNA-dependent RNA polymerase, beta' subunit (SMU_1989)		Putative endonuclease III (DNA repair) (SMU_1650)
	Recombination protein RecA (SMU_2085)		Putative DNA polymerase III, delta subunit (SMU_1662)

Transcription	DNA-dependent RNA polymerase sigma subunit; major sigma factor (sigma 70/42) (SMU_822)	DNA-dependent RNA polymerase, beta' subunit (SMU_1989)	Conserved hypothetical protein, DNA-directed RNA polymerase subunit delta (SMU_1936c)
	Putative tRNA isopentenylpyrophosphate transferase, tRNA dimethylallyltransferase (SMU_1477)		
Putative substrates (proteins with one or more predicted transmembrane domains)	Putative septation ring formation regulator (SMU_1276c)	Hypothetical protein (SMU_591c)*	Conserved hypothetical protein peptide ABC transporter substrate-binding protein (SMU_1447c)
	Putative ABC transporter, permease protein (SMU_1007)	Serine protease HtrA (SMU_2164)	Putative carbonic anhydrase precursor (SMU_1595)
	Putative PTS system, fructose-specific enzyme IIABC component (SMU_872)	Putative PTS system, mannose-specific component IID (SMU_1879)	Putative deacetylase (SMU_623c)
	Hypothetical protein (SMU_591c)*	Hemolysin (SMU_1693)	Conserved hypothetical protein, glycosyl transferase (SMU_834)
	Putative ABC transporter, ATP-binding protein ComA (SMU_286)	Hypothetical protein APQ13_07375 (ParE_toxin) (SMU_40)	Putative PTS system, glucose-specific IIABC component (SMU_2047)§
	Conserved hypothetical protein, protease (SMU_235)		RgpH (SMU_832)
	Putative cell division protein FtsH (SMU_15)§		Putative amino acid ABC transporter, periplasmic amino acid-binding protein (SMU_933)
	Putative PTS system, glucose-specific IIABC component (SMU_2047)§		Conserved hypothetical protein, cyclic nucleotide-binding protein (SMU_1307c)
	Putative PTS system, trehalose-specific IIABC component (SMU_2038)		Putative cell shape-determining protein MreC (SMU_20)
			Putative glycosyltransferase, RgpI (SMU_833)
			Conserved hypothetical protein (SMU_1477c)
			Putative undecaprenyl-phosphate-UDP- MurNAc-pentapeptide transferase (SMU_456)
			Putative cell division protein RodA (SMU_1279c)
			Integral membrane protein possibly involved in d-alanine export, d-alanyl-lipoteichoic acid biosynthesis protein DltB (SMU_1690)
			Putative transmembrane protein, permease OppC (SMU_257)
			Conserved hypothetical protein (SMU_1111c)
			Putative amino acid permease (SMU_1450)
			Hypothetical protein (SMU_503c)
			Hypothetical protein (SMU_1249c)
			Putative preprotein translocase SecY protein (SMU_2006)
			Putative sodium/amino acid (alanine) symporter (SMU_1175)
			Putative glycosyl transferase *N*-acetylglucosaminyltransferase, RgpG (SMU_246)
			Putative secreted protein, preprotein translocase subunit YajC (SMU_1787c)
			Putative endolysin, *N*-acetylmuramoyl-l-alanine amidase (SMU_707c)
			Putative manganese transporter (SMU_770c)
			Putative serine/threonine protein kinase (SMU_484)
			Putative cell division protein FtsH (SMU_15)§
			Hypothetical protein (SMU_1161c)
			Putative drug export protein; multidrug resistance protein, XRE family transcriptional regulator (SMU_745)
			Putative ABC transporter, periplasmic ferrichrome-binding protein (SMU_998)
			Cell wall-associated protein precursor WapA (SMU_2159)

a Proteins present in both upper and middle molecular weight gel slices (*) and proteins detected in regions reactive with both anti-YidC1 and anti-YidC2 antibodies (§) are indicated. PTS, phosphotransferase system.

That the upper gel-shifted region was recognized by anti-FtsY, anti-Ffh, and anti-YidC2, but not by anti-YidC1, antibodies reinforces previous genetic studies that suggest that YidC2 more so than YidC1 works in concert with the SRP pathway. Identification of Ffh and FtsY, as well as YidC2, in the upper region of the gel following whole-cell formaldehyde cross-linking is consistent with results reported in E. coli in which YidC cross-linked with Ffh and FtsY in an *in vitro* assay (18). In contrast, the middle and lower regions of the gel were reactive with both anti-YidC1 and anti-YidC2 antibodies or only with anti-YidC1 antibody, respectively, but did not react with anti-Ffh or anti-FtsY antibodies. This result again suggests that the SRP pathway may not normally function in concert with YidC1. Because S. mutans survives deletion of *yidC2*, but not of *yidC1* and *yidC2* (7), YidC1 may cooperate with the SRP pathway only as a backup mechanism when YidC2 is absent. Our previous membrane proteomic analysis of S. mutans protein transport mutants suggested that the SRP pathway acts in concert with at least one YidC paralog in the insertion of multiple substrates (10). However, that study utilized deletion mutant strains, while the current study evaluated potential protein interactions in the wild-type strain. E. coli YidC has also been shown to interact with SecYEG and that interaction is modulated by YajC (16, 18, 27, 28). The identification of SecY, YajC, and YidC1, but not YidC2, in the lower molecular weight region suggests that YidC1, rather than YidC2, functions as the default interaction partner of SecYEG-YajC holotranslocon components in S. mutans. In E. coli, the SRP receptor FtsY binds to both SecYEG and YidC; however, its affinity appears higher for SecYEG than YidC (18, 19, 29). In our cross-linking assay, FtsY colocalized with YidC2, but not with SecYEG or YidC1. FtsY interaction studies in E. coli suggest that FtsY and SecY bind to YidC at the same locus. Therefore, we speculate that dual YidC paralogs may reduce competition between SecY and FtsY in S. mutans by having SecY interact preferentially with YidC1 and YidC2 preferentially with FtsY/Ffh.

It is interesting that the membrane-associated SecA molecular motor of the general secretion pathway (30, 31) was identified in the upper molecular weight region recognized by anti-Ffh, anti-FtsY, and anti-YidC2 antibodies, but not in the lower region that contained SecY and YidC1. One reason for detection of SecA in a section of the gel reactive with anti-SRP antibodies may be through indirect bridging via ribosomal proteins. SecA and the SRP have been reported to bind to the same location on the E. coli ribosome in order to sort cellular proteins into distinct pathways for secretion through or insertion into the membrane at the site of translation (32). Our experiment utilized whole bacterial cell lysates and was skewed towards identification of cytoplasmic or membrane proteins and not expected to identify secreted SecA substrates, although SecA itself would be in proximity of SRP components on the ribosome. Also of interest, the middle molecular weight region reactive with both anti-YidC1 and anti-YidC2 antibodies did not include SecY, YajC, or SRP components. E. coli YidC is also known to function in a YidC-only pathway in the insertion of small membrane proteins (14, 23, 33). Accordingly, four out of the five membrane proteins with only one or two TM domains were detected in the middle region (Table 1). This suggests that YidC1 and/or YidC2 can work independently of both the Sec translocon and the SRP pathway in the insertion of a limited number of small membrane proteins.

RopA, the streptococcal homolog of the ribosome-associated chaperone trigger factor, was identified in both the upper and lower molecular weight regions that appear to correspond to SRP-YidC2 and SecYEG/YajC-YidC1-mediated protein translocation pathways, respectively. Trigger factor has been reported to bind to the same ribosomal protein at the peptide exit site as the SRP pathway (34, 35); therefore, the finding of RopA and Ffh in the same gel slice was not unexpected. A high proportion of the proteins (30/65) detected in the upper molecular weight region with YidC2, Ffh, and FtsY are involved in DNA replication/repair, transcription, translation, and cell division/cell shape (Table 1). Fewer such proteins colocalized with YidC1, SecY, and YajC in the lower molecular weight gel slice, and only scant ribosomal proteins colocalized with YidC1 and YidC2 in the middle molecular weight region (Table 1). These results support the idea that the YidC2-SRP cotranslational translocation pathway in particular operates in the context of a larger consortium of proteins involved in replication, transcription, and cell division. YidC1, YidC2, Ffh, and FtsY were present in both cross-linked and non-cross-linked samples as evidenced by their detection by Western blotting, even though they were not identified by mass spectrometry in these samples. Western blotting with high-quality antibodies can be more sensitive than standard bottom-up MS in the detection of certain proteins of low abundance (36).

Taken together, the results of *in vivo* whole-cell cross-linking experiments suggest three distinct patterns of association of translocation machinery components: YidC1-SecY/YajC, YidC1/2, and YidC2-SRP. To begin to identify potential substrates of these putative pathways, those proteins predicted to have one or more transmembrane domains were tabulated (Table 1). Proteins in the upper molecular weight region, suggestive of insertion by a YidC2-SRP pathway, included multiple sugar transporters and several ABC transporters, including the competence-associated protein ComA. S. mutans deletion mutants lacking *ffh* or *yidC2* exhibit seriously impaired genetic competence, while this property is notably less impacted by elimination of *yidC1* (37). We did not observe a preference for single TM compared to multipass membrane proteins in the gel slices containing YidC1/SecY/YajC or YidC2/Ffh/FtsY. However, membrane proteins from the middle molecular weight region, which likely represent substrates of a YidC1- and/or YidC2-only pathway, were mostly single- or double-pass membrane proteins except for hemolysin (SMU_1693) that contains four predicted TM domains. The identification of a higher proportion of membrane proteins in the lower molecular weight region suggests that the YidC1-SecY/YajC pathway is widely used for membrane protein insertion. Thus, YidC1 likely represents the “housekeeping” paralog in S. mutans. Membrane proteins detected in the lower molecular weight region included known or putative metal transporters, including an Nramp type Mn^2+^ transporter (SMU_770C), an iron transporter (SMU_998), and a putative zinc ABC transporter ATP-binding protein (SMU_1994), suggesting that these particular metal transporters utilize the YidC1-SecY/YajC pathway for insertion. Membrane-localized proteins related to the rhamnose-glucose polysaccharide (RGP) biosynthetic pathway, including RgpB, -G, -H, and -I were identified in the lower gel slice, suggesting them as potential YidC1 substrates. Rgp proteins, including RgpA, -B, -C, -D, -E, -F, -G, -H, and -I, were previously identified during proteomic characterization of S. mutans membrane samples (10) and variously impacted by elimination of YidC1, YidC2, or Ffh (Table 2). Of these proteins, RgpA, -C, -G, -H, and -I contain at least one transmembrane domain. The RgpB and G proteins, which colocalized with YidC1 in the lower gel slice, were absent from the Δ*ffh* Δ*yidC1* mutant and diminished in the Δ*ffh* and Δ*yidC1* single mutants, but not the Δ*yidC2* mutant. While not detected in the current interactome experiments, other Rgp proteins, including RgpD, -E, and -F were also negatively impacted by elimination of *yidC1* and *ffh* (Table 2). Impaired RGP synthesis and maturation result in decreased tolerance to bacitracin, a cyclic polypeptide antibiotic to which S. mutans is generally resistant (38). Therefore, as discussed below, we hypothesized that the Δ*yidC1*, Δ*yidC1* Δ*ffh*, and Δ*ffh* mutants, but not the Δ*yidC2* mutant, would likely be susceptible to bacitracin due to impaired RGP biosynthesis/maturation.

**TABLE 2:** Average protein scores of putative bacitracin resistance-related Rgp proteins in WT and mutant strains^*a*^

Protein	Gene	Avg protein score (PEP SUM score) ± SD				
		WT	Δ*ffh*	Δ*yidC1*	Δ*yidC2*	Δ*ffh* Δ*yidC1*
Putative RgpAc; glycosyltransferase‡	*rgpA*	6.05 ± 0.73	3.95 ± 3.3	7.24 ± 2.09	4.57 ± 1.6	3.59 ± 1.34***
Rhamnosyltransferase†	*rgpB*	1.74 ± 0.87	0.669 ± 0.668	1.23 ± 1.18	2.55 ± 2	0 ± 0**
Putative polysaccharide ABC transporter, permease protein‡	*rgpC*	1.864 ± 1.3	2.82 ± 1.75	3.18 ± 1.57	3.59 ± 1.12	1.72 ± 1.06
Putative polysaccharide ABC transporter, ATP-binding protein	*rgpD*	13.05 ± 0.69	11.71 ± 2.16	12.63 ± 1.5	11.42 ± 1.43	2.25 ± 1.61*
Putative glycosyltransferase†	*rgpE*	12.53 ± 0.64	8.29 ± 2.27**	11.78 ± 1.64	12.57 ± 1.11	3.83 ± 0.38*
RgpFc protein	*rgpF*	11.9 ± 3.33	6.17 ± 3.74***	13.45 ± 1.15	12.98 ± 1.68	3.74 ± 1.65**
Putative glycosyl transferase *N*-acetylglucosaminyltransferase), RgpG‡†	*rgpG*	1.47 ± 0.84	0 ± 0	1.01 ± 0.63	1.59 ± 0.54	0 ± 0**
Hypothetical protein (SMU_832)‡†	*rgpH*	0.96 ± 0.77	1.49 ± 0.76	2 ± 1.26	0.61 ± 0.55	0.69 ± 0.63
Putative glycosyltransferase (SMU_833)‡†	*rgpI*	4.91 ± 0.84	6.9 ± 2.25	4.45 ± 0.55	8.8 ± 1.66	3.25 ± 1.308***

a Average protein scores were determined from five replicates for each strain. Protein scores were sourced from supplemental data of Mishra et al. (10). Proteins predicted to contain one or more transmembrane domains by TMHMM v.2 (‡) and proteins detected in the current interactome analysis (†) are indicated. Statistical significance was determined by a pairwise comparison between WT and mutant strains using Student’s *t* test and indicated as follows: ***, *P* < 0.05; **, *P* < 0.005; *, *P* < 0.00001.

### Elimination of YidC1 and SRP components increases susceptibility to bacitracin.

**FIG 2:**
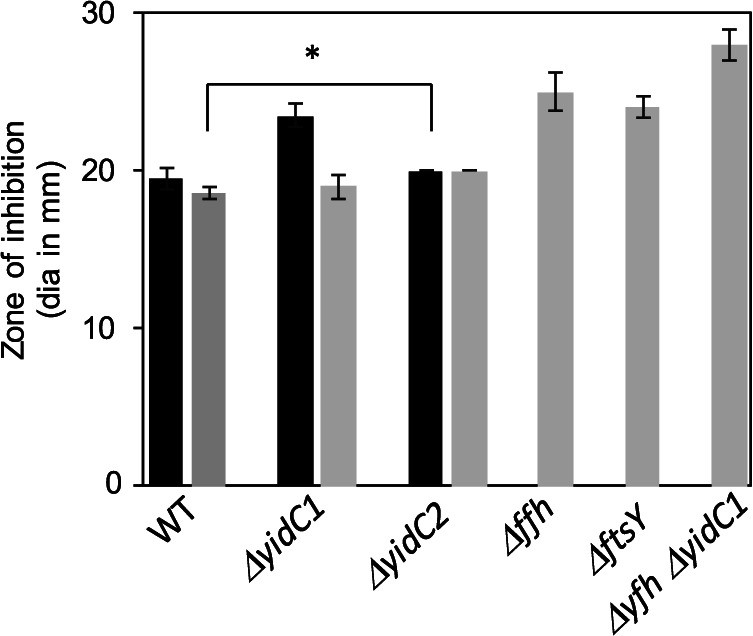
Sensitivity of wild-type and mutant strains to bacitracin. Results represent the diameter (dia) of zones of inhibition in millimeters, including the filter disc. Black bars represent UA159 or a UA159 parental background, while gray bars represent NG8 or an NG8 parental background. Mean values ± standard deviations (SD) (error bars) from three independent experiments, each performed in duplicate, are shown. Statistical significance was determined by a pairwise comparison between respective WT and mutant strains using two-tailed Student’s *t* test. *, *P* < 0.001.

RGP is distinctively associated with the outermost surface-exposed layer of S. mutans (39) and constitutes ∼50% of the total weight of the cell wall (40). S. mutans is generally resistant to bacitracin; however, disruption of *rgp* genes increases susceptibility to this antibiotic (41), because it targets the lipid carrier, C_55_-isoprenyl phosphate (IP), onto which RGP is built (38, 42). Sensitivity to 50 U of bacitracin was assessed by measuring zones of inhibition in two wild-type (WT) S. mutans strains (UA159 and NG8) as well as previously generated Δ*yidC1*, Δ*yidC2*, Δ*ffh*, Δ*ftsY*, and Δ*yidC1* Δ*ffh* mutant strains (Fig. 2) (7, 8). A Δ*yidC1* mutant demonstrated a modestly larger zone of inhibition (*P* < 0.0002) compared to its UA159 parent, but this effect was not observed in the NG8 parental background. The impact of eliminating YidC1 was much more obvious when combined with elimination of the SRP component Ffh in strain NG8. Mutant strains lacking either Ffh or the SRP receptor FtsY were also more sensitive to bacitracin than the WT, but not as much as a Δ*yidC1* Δ*ffh* double mutant. These results suggest that a cooperative pathway involving both YidC1 and the SRP contributes to bacitracin resistance in S. mutans. Hence, the *in vivo* whole-cell cross-linking approach was successful in identifying putative YidC substrates that were confirmable by predicted phenotypic properties.

### Dynabead immunocapture of protein complexes from S. mutans lysates using anti-YidC2 antibodies.

**FIG 3:**
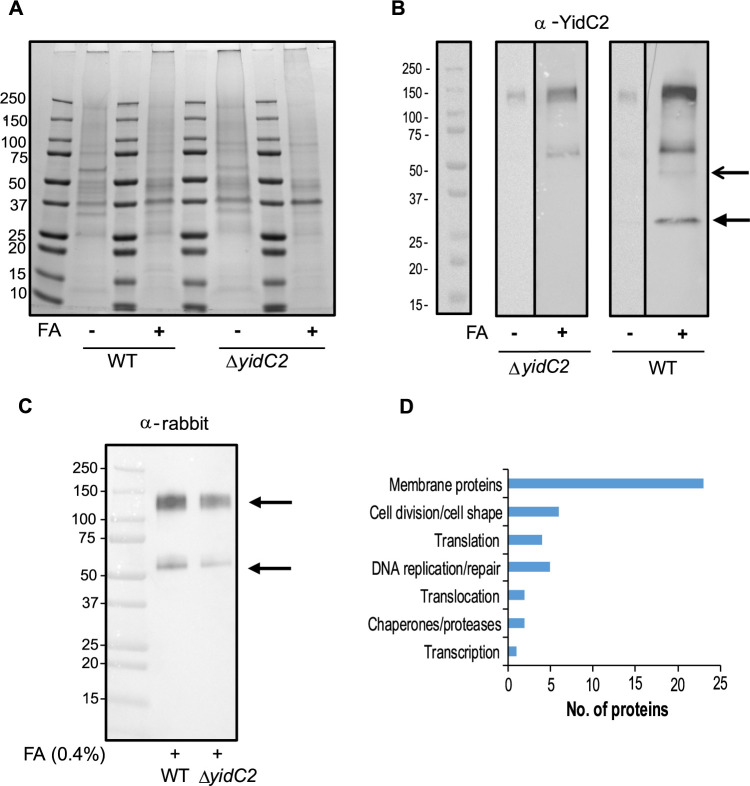
Immunocapture of YidC2 and associated protein complexes from whole-cell lysates (WCL) of S. mutans using anti-YidC2 antibodies coupled to Dynabeads. (A) SDS-PAGE. Dynabeads conjugated with anti-YidC2 antibodies were reacted with whole-cell lysates from untreated (−) or 0.4% formaldehyde cross-linked (+) wild-type S. mutans strain NG8 (WT) or corresponding Δ*yidC2* mutant and eluted with 0.5 N NH_4_OH and 0.5 mM EDTA. Migration of molecular weight standards is indicated in kilodaltons. (B) Western blot of samples shown in panel A. Bottom arrow indicates YidC2. Top arrow indicates the gel-shifted band seen only in the cross-linked sample from the WT strain. This region was excised for each of the four samples from the Coomassie blue gel, stained, and subjected to mass spectrometry analysis. (C) A replicate negative-control Western blot probed with goat anti-rabbit secondary antibodies identifies the migration of anti-YidC2 antibodies that leached from the column during the elution step. (D) Histogram showing categories of proteins cocaptured with YidC2 from the WT strain.

Because a whole-cell cross-linking approach is limited by accessibility of exposed functional groups in the target proteins to formaldehyde (43), integral membrane substrates of the insertion machinery were potentially underrepresented in our data set due to being buried within the membrane and inaccessible to the cross-linking reagent. Therefore to improve sensitivity, we also performed immunocapture experiments. To identify potential YidC2 binding partners, anti-YidC2 antibodies were covalently coupled to magnetic Dynabeads, which reacted with whole-cell lysates from untreated and formaldehyde-treated cells of S. mutans wild-type strain NG8 and its corresponding Δ*yidC2* mutant. The two polyclonal rabbit antisera used were raised against synthetic peptides corresponding to the YidC2 C-terminal tail and to the cytoplasmic loop between TM2 and TM3. Bound proteins were eluted from the antibody-coupled Dynabeads with glycine-HCl (pH 2.0). Aliquots of each sample were analyzed by Western blotting (Fig. 3A). As expected, a 27-kDa YidC2 band was identified in the wild type, but not in the Δ*yidC2* strain (Fig. 3B). An additional ∼45-kDa band reactive with anti-YidC2 antibodies was also observed in the cross-linked WT sample, but not samples from the mutant strain or the non-cross-linked control sample from the WT strain. Higher-molecular-weight bands observed in the replicate negative-control blot probed only with goat anti-rabbit heavy-chain-specific secondary antibodies represented anti-YidC2 antibodies that leached from the Dynabeads (Fig. 3C). Gel slices corresponding to the ∼45-kDa region of interest identified by Western blotting were cut from SDS-polyacylamide gels of all four samples and analyzed by MS. A total of 269 proteins were identified in the WT cross-linked sample, 229 in the WT non-cross-linked sample, 246 proteins in the Δ*yidC2* cross-linked sample, and 284 proteins in the Δ*yidC2* non-cross-linked sample. Sixty-eight proteins were present in the WT cross-linked sample and 31 proteins in the non-cross-linked sample that were absent from the corresponding Δ*yidC2* samples (summarized in Table S2). Twenty-five percent of the proteins contained one or more TM domains (TMDs), and the remainder were shown to be membrane associated based on previous membrane proteomic analysis (10). Figure 3D shows a graphical representation of the types of proteins that were cocaptured with YidC2.

10.1128/mSphere.01308-20.6 TABLE S2 Summary of proteins captured with anti-YidC2 antibodies from formaldehyde-cross-linked and non-cross-linked S. mutans wild-type (WT) whole-cell lysates compared to the Δ*yidC2* mutant strain. Download Table S2, PDF file, 0.5 MB. Copyright © 2021 Vasquez et al. 2021 Vasquez et al. https://creativecommons.org/licenses/by/4.0/ This content is distributed under the terms of the Creative Commons Attribution 4.0 International license.

Potential YidC2 substrates identified by this immunocapture experiment included two subunits of the phosphotransferase system (PTS) mannose transporter, metalloprotease (RseP), histidine kinases, enzymes, and cell wall/cell division- related proteins (Table 3). In agreement with our prior gel shift experiment, we identified SecA as well as other proteins involved in DNA replication/repair, transcription, translation, and cell division/cell shape in association with YidC2 (Fig. 3D). Again, this suggests that translocation is part of a coordinated machinery that incorporates additional processes beyond protein translation. While the YidC2 cross-linked sample contained YajC, other members of the holotranslocon were not cocaptured with YidC2. Collectively, our anti-YidC2 immunocapture assay identified translocation machinery components, ribosomal proteins, chaperones, and proteases, enzymes involved in DNA replication and repair, and proteins responsible for cell wall generation and cell division. Because of modest coupling efficiency of Dynabeads with anti-YidC2 antibodies, this approach was not extended to anti-YidC1 antibodies.

**TABLE 3:** Functional categories of proteins captured with anti-YidC2 coupled Dynabeads from S. mutans whole-cell lysates but absent from Δ*yidC2* mutant strain samples

Functional category and protein^*a*^
Translation
50S ribosomal protein L14 (SMU_2017)
50S ribosomal protein L31 type B (SMU_1298)
50S ribosomal protein L16 (SMU_2020)
tRNA uridine 5-carboxymethylaminomethyl modification protein (SMU_2141)
Translocation
Preprotein translocase subunit SecA (SMU_1838)
Preprotein translocase subunit YajC (SMU_1787c)
DNA replication/repair
DNA-directed RNA polymerase subunit beta' (SMU_1989)
DNA repair protein RadA (SMU_327)
Deoxyribonuclease HsdR
DNA topoisomerase I (SMU_1002)
DNA topoisomerase IV subunit B (SMU_1277)
Chaperones/proteases
Metalloprotease RseP* (SMU_1784c)
ATP-dependent Clp protease ATP-binding subunit (SMU_956)
Cell wall/cell shape/cell division
Penicillin-binding protein Pbp2b (SMU_597)
Peptidoglycan branched peptide synthesis protein MurM* (SMU_716)
Septation ring formation regulator EzrA (SMU_1276c)
d-Alanyl-lipoteichoic acid biosynthesis protein DltD (SMU_1688)
Rod shape-determining protein RodA (SMU_1279c)
Cell division protein SepF (SMU_554)
Putative substrates (with one or more predicted TM domains)
Penicillin-binding protein (SMU_597)
Metalloprotease RseP* (SMU_1784c)
Septation ring formation regulator EzrA (SMU_1276c)
Cytoplasmic membrane protein, LemA (SMU_1930)
d-Alanyl-lipoteichoic acid biosynthesis protein DltD (SMU_1688)
Serine/threonine protein kinase (SMU_484)
Preprotein translocase subunit YajC (SMU_1787c)
PTS mannose family transporter subunit IID (SMU_1879)
Hypothetical protein APQ13_00045 (SMU_1719c)
Acyltransferase (SMU_67)
ABC transporter permease (SMU_396)
Hypothetical protein APQ13_06235 (SMU_333)
Hypothetical protein APQ13_07285 (SMU_66)
PTS mannose transporter subunit IIC (SMU_1878)
Hemolysin (SMU_1693)
Phosphatidate cytidylyltransferase (SMU_1785)
Murein hydrolase transporter LrgA (SMU_575c)
Glycosidase
Hypothetical protein APQ13_09110 (SMU_1856c)
Rod shape-determining protein RodA (SMU_1279c)
Hypothetical protein APQ13_07320
PAS domain-containing sensor histidine kinase (SMU_1516)
Histidine kinase (SMU_1145c)

a Proteins present in both formaldehyde-cross-linked and non-cross-linked samples are indicated by an asterisk.

### Difference gel electrophoresis of S. mutans proteins captured by YidC1 or YidC2 C-terminal tails.

**FIG 4:**
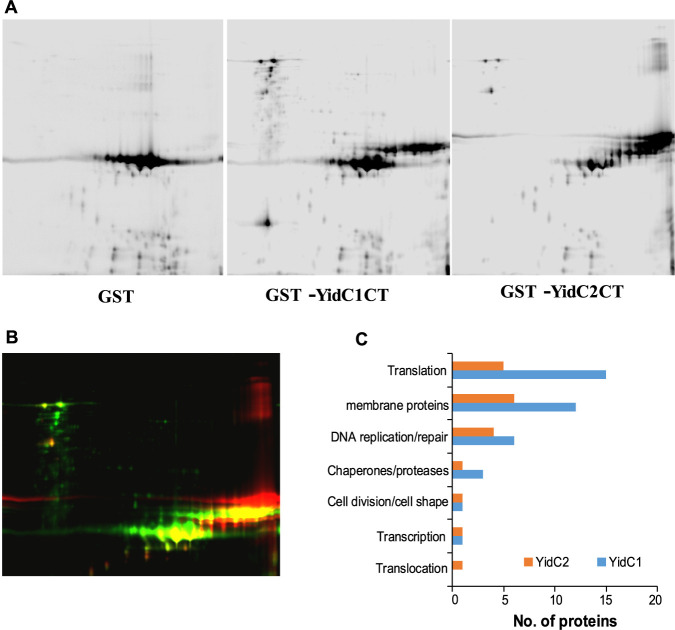
Proteins cocaptured with GST, GST-YidC1CT, or GST-YidC2CT analyzed by 2D-DIGE. (A) S. mutans whole-cell lysates were reacted with the indicated GST polypeptide and captured using glutathione affinity chromatography. The eluted samples were labeled with CyDye DIGE fluors (YidC1CT with red Cy3, YidC2CT with green Cy2, and GST with blue Cy5) and separated on a single 2D gel by isoelectric focusing in the first dimension and SDS-PAGE in the second dimension. Black and white images for each sample are shown. (B) Signals from each dye were scanned, and the three images were overlaid. One hundred twenty separate spots (shown in Fig. S2 in the supplemental material) were excised from the gel for mass spectrometry analysis. (C) The numbers and types of proteins associated with GST-YIDC1CT compared to GST-YidC2CT are shown.

As an alternative to immobilization of anti-YidC antibodies to Dynabeads, we also utilized a glutathione-*S*-transferase (GST) tag-based pulldown approach. While it is difficult to express full-length S. mutans *yidC1* and *yidC2* in E. coli to sufficient levels for large-scale protein purification, the YidC1 and YidC2 C-terminal tails are both soluble, and easily tagged and purified. We constructed fusion proteins of the YidC1 and YidC2 C-terminal tail domains with GST and affinity purified the recombinant polypeptides on glutathione Sepharose (see Fig. S1 in the supplemental material). Because domain swapping experiments have demonstrated that the positively charged tails of S. mutans YidC1 and YidC2 contribute to certain functional attributes of each paralog (8), we expected a subset of YidC1 and YidC2 binding partners to interact with these domains. After purification, the GST-YidC1-tail and GST-YidC2-tail fusion proteins were reacted with S. mutans whole-cell lysates (non-cross-linked) and captured on immobilized glutathione using GST as a negative control. Following elution with reduced glutathione, the three samples were individually labeled with a different CyDye fluorescent dye and subjected to 2D-difference gel electrophoresis (DIGE) (Fig. 4). One hundred twenty-one spots were identified as being captured by GST-YidC1CT and/or GST-YidC2CT, but not by GST alone (Fig. S2). A complete list of all proteins identified in each of the gel spots is shown in Table S3. A summary of the proteins pulled down with GST-Yid1CT (green spots), GST-YidC2CT (red spots), or both (yellow spots) is shown in Table S4. Seventy-four proteins were cocaptured with GST-YidC1CT, and 37 were cocaptured with GST-YidC2CT (Table S3). Of these proteins, 42 were uniquely cocaptured with GST-YidC1CT, while only 5 were uniquely cocaptured with GST-YidC2CT (Table S4).

10.1128/mSphere.01308-20.2 FIG S1 Preparation of samples for difference gel electrophoresis of S. mutans proteins captured by YidC1 or YidC2 C-terminal tails. (A) SDS-PAGE of purified GST-YidC1CT, GST-YidC2CT, and GST polypeptides. The GST-YidC1CT fusion construct is prone to breakdown to GST (marked with an asterisk). (B) S. mutans whole-cell lysates were reacted with the GST polypeptides, captured on glutathione-Sepharose, eluted with reduced glutathione, and visualized by SDS-PAGE. Download FIG S1, PDF file, 0.6 MB. Copyright © 2021 Vasquez et al. 2021 Vasquez et al. https://creativecommons.org/licenses/by/4.0/ This content is distributed under the terms of the Creative Commons Attribution 4.0 International license.

10.1128/mSphere.01308-20.3 FIG S2 Proteins cocaptured with GST, GST-YidC1CT, or GST-YidC2CT analyzed by 2D-DIGE. S. mutans whole-cell lysates were reacted with the indicated GST polypeptide and captured using glutathione affinity chromatography (Fig. S1). The eluted samples were labeled with CyDye DIGE fluors (YidC1CT with red Cy3, YidC2CT with green Cy2, and GST with blue Cy5) and separated on a single 2D gel, with isoelectric focusing in the first dimension and SDS-PAGE in the second dimension. Signals from each dye were scanned, and the three images overlaid. One hundred twenty separate spots (indicated by circles) were excised from the gel for mass spectrometry analysis. All proteins identified in each spot are listed in Table S3. Download FIG S2, PDF file, 0.5 MB. Copyright © 2021 Vasquez et al. 2021 Vasquez et al. https://creativecommons.org/licenses/by/4.0/ This content is distributed under the terms of the Creative Commons Attribution 4.0 International license.

10.1128/mSphere.01308-20.7 TABLE S3 List of proteins identified by DIGE experiment along with description of the spots. Download Table S3, XLSX file, 0.04 MB. Copyright © 2021 Vasquez et al. 2021 Vasquez et al. https://creativecommons.org/licenses/by/4.0/ This content is distributed under the terms of the Creative Commons Attribution 4.0 International license.

10.1128/mSphere.01308-20.8 TABLE S4 List of proteins identified by GST-YidC1CT and GST YidC2CT pulldown assay by 2DIGE. Download Table S4, PDF file, 0.4 MB. Copyright © 2021 Vasquez et al. 2021 Vasquez et al. https://creativecommons.org/licenses/by/4.0/ This content is distributed under the terms of the Creative Commons Attribution 4.0 International license.

The types of proteins cocaptured with GST-YidC1CT compared to GST-YidC2CT are summarized in Table 4. Eleven different integral membrane proteins were found as part of the YidC1-tail interactome, including five that were also pulled down with GST-YidC2CT. Most of these were transporters except for the cell division protein FtsH and a histidine kinase (SMU_486). All nonintegral membrane proteins identified by DIGE (∼85%) had previously been identified as being membrane associated during proteomic analysis of S. mutans protoplast-derived membrane preparations (10). The predominance of nonintegral membrane proteins in the DIGE data set suggest that the YidC1 and YidC2 C-terminal tails do not play a prominent role in recognizing and binding substrates. Interestingly, the SRP component protein Ffh was found in association with the YidC2 tail, but not with the YidC1 tail. This is consistent with data from the *in vivo* formaldehyde cross-linking experiments that suggested a preference of YidC2 instead of YidC1 in a cooperative SRP-YidC pathway. None of the components of the SecYEG translocon, nor YajC, were identified in association with either of the C-terminal tails; thus, these domains likely do not contribute to YidC1 or YidC2 interactions with the translocon components themselves. Consistent with this observation, Petriman et al. showed that TM1, TM3, TM4, the periplasmic domain, and cytoplasmic loop C1 of *E.coli* YidC interact with SecY (18).

**TABLE 4:** List of S. mutans proteins pulled down with GST-YidC1CT and/or GST-YidC2CT, but not GST, identified by 2D-DIGE and mass spectrometry

Category	GST-YidC1-CT	GST-YidC2-CT
Translocation		Signal recognition particle protein (SMU_1060)

Translation	50S ribosomal protein L2 (SMU_2160)	50S ribosomal protein L2 (SMU_2160)
	50S ribosomal protein L6 (SMU_2011)	50S ribosomal protein L6 (SMU_2011)
	50S ribosomal protein L13 (SMU_169)	50S ribosomal protein L13 (SMU_169)
	Putative ribosomal protein S1 (SMU_1200)	30S ribosomal protein S7 (SMU_358)
	30S ribosomal protein S2 (SMU_2032)	30S ribosomal protein S17 (SMU_2017)
	30S ribosomal protein S4 (SMU_2135c)	
	30S ribosomal protein S7 (SMU_358)	
	30S ribosomal protein S8 (SMU_2012)	
	30S ribosomal protein S17 (SMU_2017)	
	30S ribosomal protein S21 (SMU_818)	
	Elongation factor Tu (SMU_714)	
	Phenylalanyl-tRNA synthetase subunit alpha (SMU_1512)	
	Glycyl-tRNA synthetase subunit alpha (SMU_445)	
	S1 RNA-binding domain-containing protein (SMU_1623c)	
	tRNA (adenine (22)-N(1))-methyltransferase (SMU_1464c)	

DNA replication/repair	DNA polymerase III, gamma/tau subunit (SMU_1581)	DNA polymerase III, gamma/tau subunit (SMU_1581)
	DNA polymerase III PolC (SMU_123)	DNA polymerase III PolC (SMU_123)
	DNA-directed RNA polymerase subunit alpha (SMU_2001)	DNA-directed RNA polymerase subunit alpha (SMU_2001)
	DNA polymerase I (POL I) (SMU_297)	Holliday junction-specific endonuclease (SMU_469)
	DNA repair protein RecN (SMU_585)	
	DNA mismatch repair protein MutS (SMU_2091c)	

Transcription	Probable DNA-directed RNA polymerase subunit delta (SMU_96)	Probable DNA-directed RNA polymerase subunit delta (SMU_96)

Chaperones/proteases	Molecular chaperone DnaK (SMU_82)	Molecular chaperone DnaK (SMU_82)
	Chaperone protein ClpB (SMU_1425)	
	Heat shock protein GrpE (SMU_81)	

Cell wall/cell shape/cell division	Cell division protein FtsH (SMU_15)	Cell division protein FtsH (SMU_15)

Putative substrates (with one or more predicted TM domains)	Cell division protein FtsH (SMU_15)	ABC transporter ATP-binding protein (SMU_906)
	Putative amino acid ABC transporter, permease protein (SMU_1216c)	Putative ABC transporter, substrate-binding protein (SMU_651c)
	ABC transporter ATP-binding protein (SMU_906)	Histidine kinase (SMU_486)
	Histidine kinase (SMU_486)	Cell division protein FtsH (SMU_15)
	Putative PTS system, glucose-specific IIABC (SMU_2047)	Putative amino acid ABC transporter, permease protein (SMU_1216c)
	Putative ABC transporter, substrate-binding protein (SMU_651c)	
	Dextranase (SMU_2042)	
	Hypothetical protein (SMU_791c)	
	Conserved hypothetical protein (SMU_485)	
	Potassium transporter peripheral membrane protein (SMU_1708)	
	EamA family transporter (SMU_1560)	

As described above in both gel shift and YidC2 immunocapture experiments, numerous ribosomal proteins, as well as other components of the translation machinery, were captured in association with YidC1 and/or YidC2. Such proteins were found irrespective of whether Ffh and FtsY were also present, suggesting that either S. mutans YidC paralog can act to support cotranslational protein translocation in the absence of the SRP pathway. Indeed, YidC2 was previously demonstrated to complement Oxa1 deficiency in yeast mitochondria that lack an SRP pathway (44). Although *yidC1* was also expressed in yeast cell extracts, its protein product was not properly imported into the mitochondria and therefore could not be assessed in complementation experiments (44). When overexpressed in E. coli, both S. mutans YidC1 and YidC2 were reported to interact with translating and nontranslating ribosomes by a tail-dependent mechanism (45). The large ribosomal subunit protein, L2, was the most abundant ribosomal protein pulled down by the GST-YidC1CT or GST-YidC2CT fusion polypeptides. In E. coli, L2 not only acts as a structural component of the ribosome, it is also processed to a truncated derivative (tL2) that can interact with the RNA polymerase alpha subunit and modulate transcription (46). E. coli L2 has also been reported to interact with the Hsp90 homolog HtpG to modulate its ATPase activity and also to bind to other chaperones, including DnaK/DnaJ/GrpE and GroEL/GroES (47). Full-length and truncated E. coli L2 also both interact with DnaA to modulate DNA replication (48). DNA segments encoding S. mutans L2 and truncated (trL2) were cloned, and the recombinant His-tagged proteins were tested by enzyme-linked immunosorbent assay (ELISA) to determine whether either form interacts directly with the C-terminal tails of YidC1 or YidC2. Neither L2 nor trL2 demonstrated significant binding to GST-YidC1CT or to GST-YidC2CT (Fig. S3). Likewise, SecA, which had been observed in conjunction with YidC2 in both Western blot gel shift and immunocapture experiments, did not react directly with GST-YidC2CT (or GST-YidC1CT) (Fig. S3). This suggests that the association of SecA with YidC2 is indirect or not mediated by the YidC2 tail.

10.1128/mSphere.01308-20.4 FIG S3 Assessment of potential interactions between the C-terminal tails of YidC1 or YidC2 with ribosomal protein L2, truncated L2, or SecA. ELISA plate wells were coated with 400 ng of GST-YidC1CT (A) or GST-YidC2CT (B) and then overlaid with the indicated amount of recombinant His-tagged L2, tL2, or SecA. Binding of overlaid proteins was evaluated with murine anti-His antibodies. Adequate coating of wells with GST-YidC1CT or GST-YidC2CT was confirmed using rabbit polyclonal antibodies against the C-terminal tails of YidC1 or YidC2. Download FIG S3, PDF file, 0.3 MB. Copyright © 2021 Vasquez et al. 2021 Vasquez et al. https://creativecommons.org/licenses/by/4.0/ This content is distributed under the terms of the Creative Commons Attribution 4.0 International license.

Similar to formaldehyde cross-linking and immunocapture experiments, GST-YidC1CT and GST-YidC2CT pulldowns and DIGE identified a variety of proteins, including chaperones and proteins involved in replication, transcription, translation, and cell division/cell shape, again suggesting that these processes are temporally and spatially connected. Such data are consistent with the identification of coupled transcription/translation in other bacteria, which may also integrate aspects of DNA replication (49–53). Both YidC1 and YidC2 contribute to proper cell wall biosynthesis and cell morphology in S. mutans (9); thus, capture of proteins in this category is consistent with previously described mutant phenotypes.

### Bacterial two-hybrid analysis to assess direct interactions of YidC1 and YidC2 with putative interactome members.

**FIG 5:**
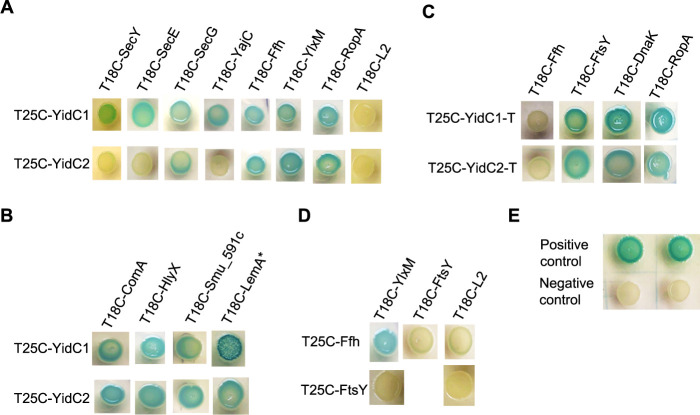
BACTH-based bacterial two-hybrid assays for direct protein-protein interaction analysis. Genes encoding indicated polypeptides were fused in frame with T25 or T18 fragments at the C terminus and expressed in E. coli *cya* mutant BTH101 cells. Polypeptides were full length, or almost full length, except for YidC1 and YidC2 C-terminal tails. Cotransformants containing bait and target expression plasmids were spotted onto LB agar containing appropriate antibiotic selection markers and substrate. (A) Assessment of interactions of YidC1 or YidC2 with indicated protein translocation machinery components, chaperone RopA (trigger factor), or ribosomal protein L2. (B) Assessment of interactions of YidC1 or YidC2 with putative substrates identified in pulldown experiments. The asterisk in T18C-LemA* indicates that an interaction with the YidC1-tail fusion was assessed. (C) Assessment of interactions of the C-terminal tails of YidC1 or YidC2 with the indicated proteins. (D) Assessment of interactions of signal recognition particle pathway components, Ffh or FtsY, with indicated protein translocation machinery components of ribosomal protein L2. (E) BACTH system controls. A strain coexpressing T25 and T18 fragments fused to the leucine zipper domain was used as the positive control. A strain containing empty pKT25-pUT18C plasmid, was used as the negative control.

Although the gel shift and pulldown screening approaches described above identified potential binding partners of YidC1 and YidC2, such experiments alone are insufficient to demonstrate direct protein-protein interactions. To evaluate specific interactions of given proteins with the full-length YidC1 and YidC2 insertases or with their C-terminal tails, we selected a subset of proteins that represent both machinery components and putative substrates. Interactions were assessed using the bacterial two-hybrid (BACTH) system (Euromedex). Proteins of interest were produced as fusion partners, generally at the C terminus, of the catalytic domain of the chimeric adenylate cyclase (T25 or T18) of Bordetella pertussis. An association between the hybrid target and the bait protein results in restoration of cyclic AMP (cAMP) production in an E. coli *cya* mutant (54). If a cAMP complex is formed with the carbon catabolite activator protein, genes of the lactose operon are expressed, resulting in a blue colony on selection plates containing substrate. Due to the potential toxicity of streptococcal membrane proteins in E. coli, the YidC1 and YidC2 targets were expressed in the low-copy-number plasmid pKT25, while the bait proteins were expressed in the high-copy-number plasmid pUT18C. To identify protein-protein interactions, plasmids with bait and target genes were cotransformed in the E. coli *cya* mutant, BTH101, and resulting colonies were examined for blue coloration (Fig. 5). Results demonstrated that YidC1 interacts directly with the holotranslocon components (SecYEG-YajC), SRP/SRP receptor proteins (Ffh, YlxM/FtsY), and chaperones (DnaK and RopA) (Fig. 5A). Notably, YidC2 did not interact with SecY, SecE, or YajC. These results are consistent with our *in vivo* whole-cell cross-linking data whereby YidC2 did not colocalize with SecY and YajC in gel shift experiments. When tested by BACTH, YidC2 interacted only with the SecG holotranslocon accessory protein. YidC1’s ability to interact with SecY, SecE, and SecG is more similar than YidC2 to the SecYEG interaction reported for E. coli YidC (18). YidC1 and YidC2 were both nonreactive with L2 when tested by BACTH (Fig. 5A), again suggesting that L2 is an indirect component of the YidC1 and YidC2 interactomes.

Potential YidC2 substrates identified in our screening experiments included the membrane proteins ComA, Smu_591c, HlyX, and LemA. These proteins interacted with both YidC1 and YidC2 when tested using the BACTH system (Fig. 5B). This suggests that while these proteins appear to represent preferred YidC2 substrates under routine growth conditions, they can also associate with YidC1, which may serve as a backup when YidC2 is absent. The potential interactions identified by DIGE of the membrane protein LemA and the chaperones DnaK and RopA with the C-terminal tails of YidC1 and YidC2 were also confirmed by the bacterial two-hybrid assay (Fig. 5C). Both YidC1 and YidC2 interacted with the SRP component Ffh by BACTH assay, but the possible interaction of Ffh with the YidC2 tail suggested by DIGE was not confirmed. It is likely, therefore, that the YidC2-tail-Ffh association is not direct and occurs within the context of a more extensive molecular complex. In fact, both the YidC1 and YidC2 C-terminal tails interacted with FtsY, a known direct binding partner of Ffh (Fig. 5C). It has been reported in E. coli that SecYEG and YidC compete for binding to the SRP receptor, FtsY (18). As expected, Ffh interacted directly by BACTH assay with the S. mutans SRP accessory component YlxM (Fig. 5A), while FtsY did not (Fig. 5D). YlxM was demonstrated previously to bind directly to Ffh and to the SRP small cytoplasmic RNA, but not to FtsY, and to modulate GTP hydrolysis in the presence of these SRP pathway components (55). We used Ffh-YlxM and FtsY-YlxM as suitable positive and negative controls based on these previously established interactions in addition to the positive- and negative-control plasmids provided with the BACTH kit (Fig. 5E). The two-hybrid assay results indicate that similar to Ffh, YlxM is also capable of direct interactions with both YidC1 and YidC2. The BACTH approach also identified direct interactions of Ffh with the holotranslocon components SecG and YajC, but not with SecE. In E. coli, FtsY has been reported to interact directly with SecY; however, our BACTH results did not identify strong clear cut interactions of FtsY with SecYEG (Fig. 5D). Taken together, the results of BACTH system assays confirmed putative interactions of YidC1 and YidC2 with multiple proteins predicted by whole-cell formaldehyde cross-linking and DIGE pulldown experiments.

### Determination of YidC1 and YidC2 interactomes and functional annotation.

**FIG 6:**
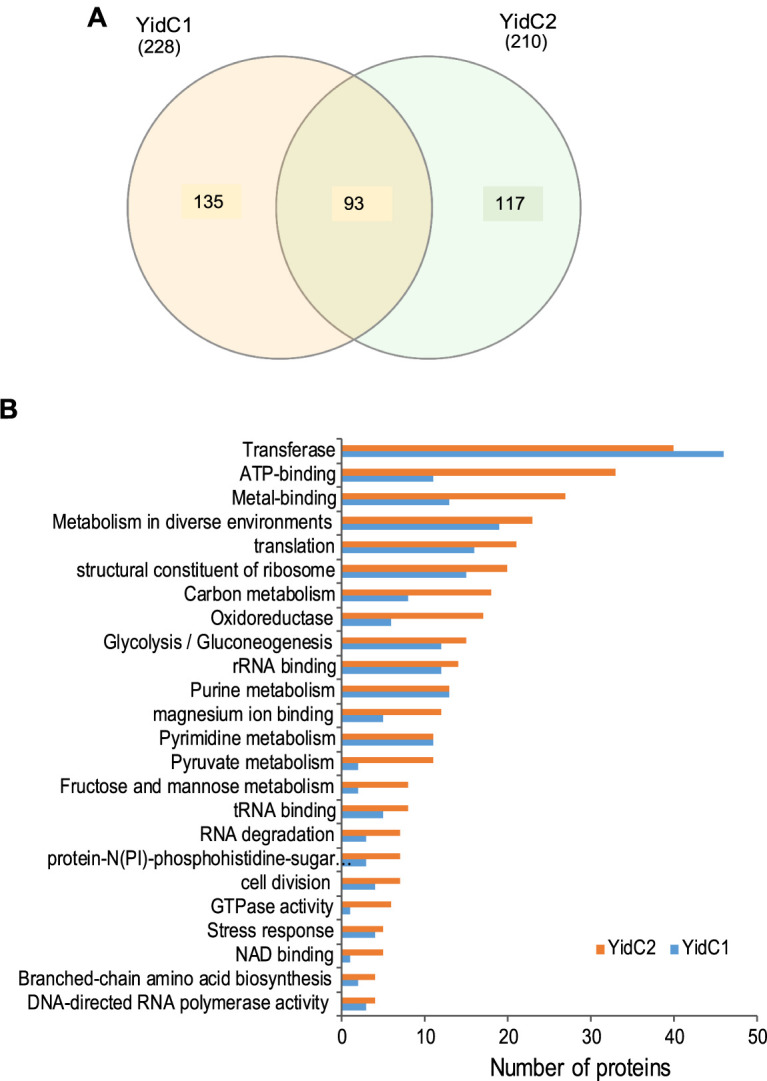
Comparison of YidC1 and YidC2 interactomes. (A) Venn diagram illustrating the degree of overlap of proteins identified in YidC1 and YidC2 interactomes. (B) Distribution of proteins within the YidC1 and YidC2 interactomes according to functional categories (DAVID analysis).

When proteins from all experiments were evaluated in composite, 93 were identified as being associated with both YidC1 and YidC2, while 135 or 117 were uniquely associated with YidC1 or YidC2, respectively (Fig. 6A). Whenever possible, proteins were assigned to functional categories by Database for Annotation, Visualization, and Integrated Discovery (DAVID) analysis (Fig. 6B). The most prevalent functional category in both interactomes was transferase. Functional annotation also showed that the YidC2 interactome, compared to the YidC1 interactome, was enriched in a number of functional categories, including ATP-binding proteins, metalloproteins, carbon metabolism, oxidoreductases, cell division, GTPase activity, and branched-chain amino acid pathways. This may explain why the phenotypic consequence of elimination of YidC2 is far more pronounced than elimination of YidC1 (7, 8, 37). In contrast, the only instances in which the YidC1 interactome equaled or exceeded that of YidC2 were in the transferase and purine and pyrimidine metabolism categories. Of note, however, a greater number of proteins in the YidC1 interactome are either not annotated or have putative individualized functions that cannot be assigned to a broad category.

**FIG 7:**
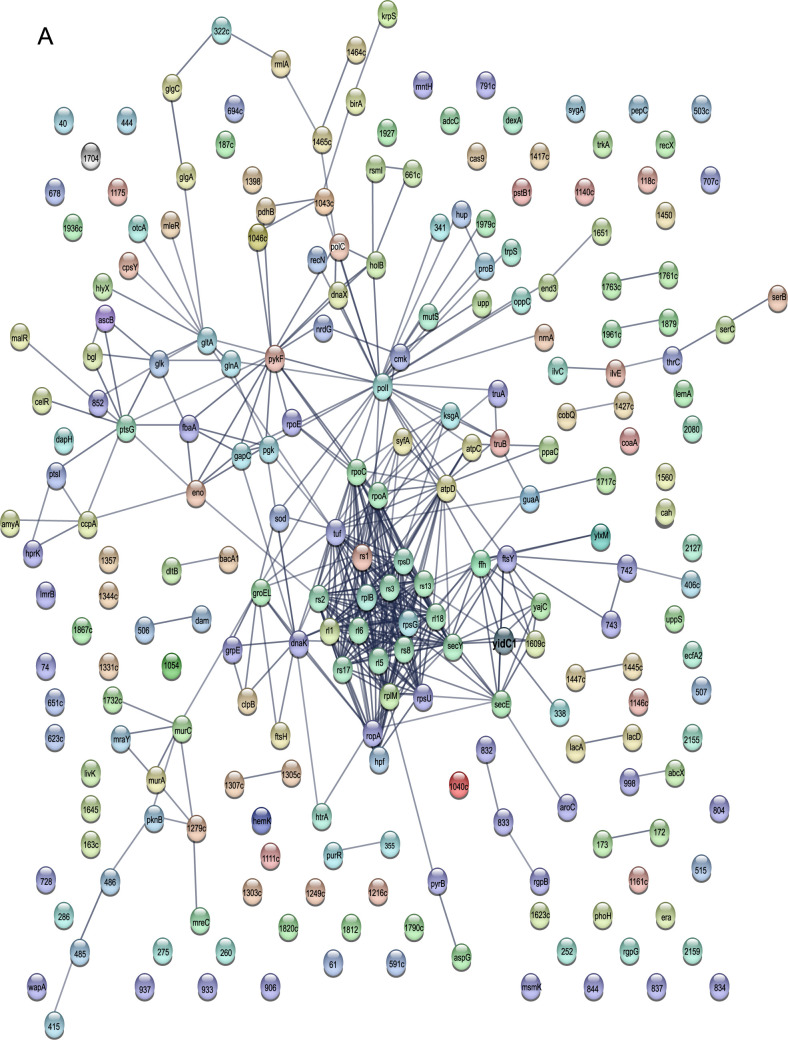

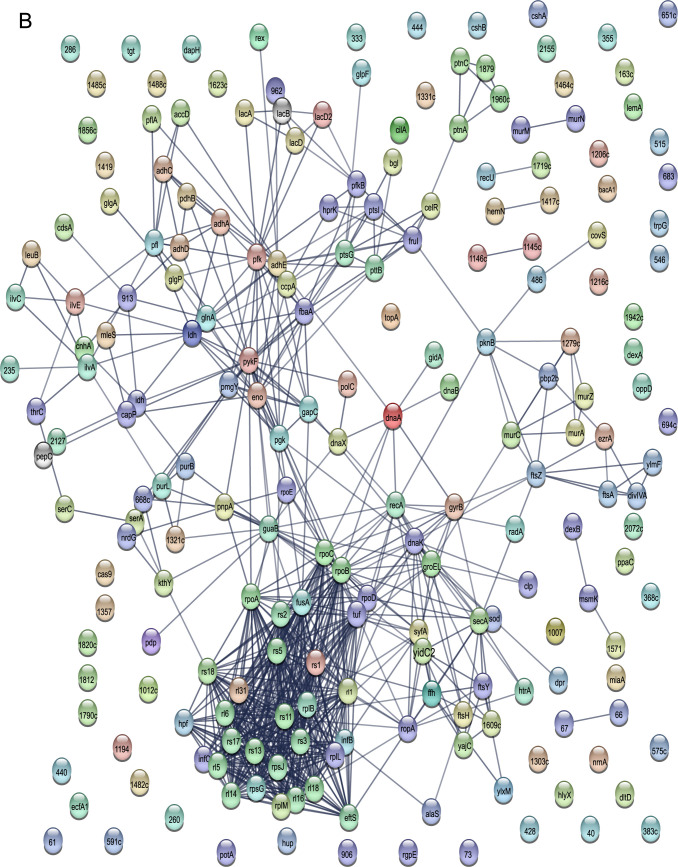

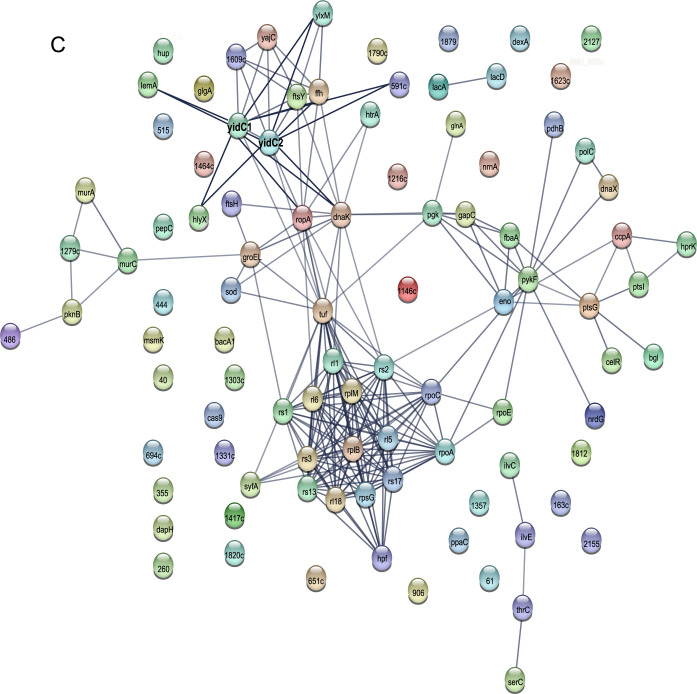
Protein-protein interaction networks predicted by STRING analysis. (A) YidC1 interactome; (B) YidC2 interactome; (C) YidC1 and YidC2 shared interactome. Each protein experimentally determined in the current study to associate with YidC1 and/or YidC2 is depicted by a sphere with either the protein name or SMU number indicated. YidC1 and YidC2 are indicated in boldface type. Lines indicate predicted interactions based on current information within the STRING database.

We also carried out protein-protein interaction (PPI) network analysis using the STRING (Search Tool for the Retrieval of Interacting Genes/Proteins) (56). YidC1 and YidC2, as well as all proteins experimentally identified in association with either or both of these insertases, were included in the uploaded data sets. The individual YidC1 and YidC2 STRING interactomes are shown in Fig. 7A and B, and the common interactome is shown in Fig. 7C. Approximately 70% of the proteins we identified in the current study were included in the PPI networks predicted by STRING, thus giving us high confidence in the accuracy of the experimentally determined protein interactomes. Consistent with cotranslational protein translocation pathways, the most intense nodes identified in all three PPI network predictions were largely comprised of ribosomal proteins and other components of the translation machinery. L2, which we determined by ELISA not to interact with the YidC1 or YidC2 C-terminal tails (Fig. S3), was not predicted by STRING analysis to interact with either insertase. S1, however, is a predicted STRING interaction partner of L2, as well as of YidC1 and YidC2. S1 is therefore a likely bridging molecule, since it was detected experimentally whenever L2 was found in association with either YidC1 or YidC2.

### Concluding remarks and apparent S. mutans protein translocation pathways.

**FIG 8:**
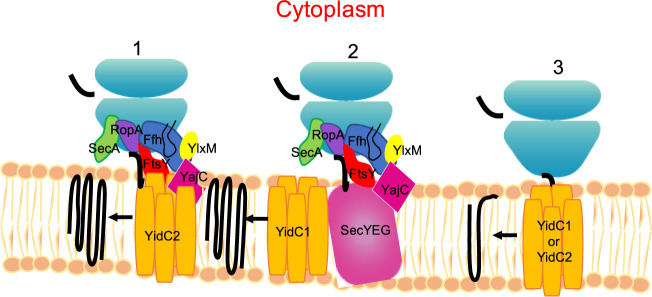
Model representation of putative cotranslational membrane protein insertion pathways in S. mutans. (Pathway 1) SRP-YidC2 pathway. YidC2 works in concert with the signal recognition particle (SRP) pathway. The SRP is comprised of Ffh, a small cytoplasmic RNA, and the YlxM accessory protein present only in Gram-positive bacteria (55). The SRP targets the ribosome nascent chain complex to the membrane via a reversible interaction of Ffh with the SRP receptor FtsY. The substrate protein is then passed to YidC2 from FtsY for integration into the membrane. RopA and SecA fractionate with components of this pathway because of their common association with large ribosomal subunit proteins. (Pathway 2) SecY-YidC1 pathway. Integral membrane proteins are targeted to SecYEG with the help of FtsY, RopA, and other chaperones (DnaK, GroEL), and insertion into the membrane is facilitated by YidC1. (Pathway 3) YidC1 and/or YidC2 autonomous pathway. A small subset of membrane proteins having one or two transmembrane domains can be inserted into the membrane independently of SecYEG or the SRP.

Most information regarding bacterial membrane protein translocation comes from the Gram-negative bacterium E. coli; however, Gram-positive bacteria generally have two YidCs, and based on genomic sequences, a seemingly smaller holotranslocon whereby SecDF are lacking in streptococci. Existing literature reports from E. coli suggest that most membrane proteins are translocated in a SRP-dependent manner via the SecYEG translocon and/or YidC (4). Nevertheless, a small number of membrane proteins have been shown to be translocated in a YidC-dependent, but SRP- and SecYEG-independent manner (22, 57–59). More recently, an SRP-dependent posttranslational pathway of translocation for the small membrane protein YohP was also identified in E. coli (60). Our current results suggest that S. mutans utilizes similar pathways, with YidC1 preferentially associating with the Sec translocon in the absence of the SRP, and YidC2 being the preferred insertase when the SRP pathway is employed (Fig. 8). Additionally, a separate YidC1 and/or YidC2-only membrane insertion pathway appears to function independently of both the SRP pathway and SecYEG. BACTH analysis showed a direct interaction of FtsY with the C-terminal tails of YidC1 and YidC2 tails, but not with SecYEG (Fig. 5D) We also identified interactions of Ffh with full-length YidC1, YidC2, and the holotranslocon components SecG and YajC, suggesting that partitioning into SecYEG/YidC1 and SRP/YidC2 can be likely achieved through SRP-YidC1/2 interaction. Unlike the E. coli holotranslocon, which consists of SecYEG-SecDF-YajC-YidC, the S. mutans holotranslocon appears to consist of SecYEG-YajC-YidC1, while the presence of YidC2 remains elusive.

Because Δ*yidC2* and SRP pathway mutants are similarly impaired growth during acid, oxidative, or osmotic stress (7), the cooperative YidC2-SRP pathway appears to be particularly important in the insertion of substrates that confer stress tolerance. All of the current experiments were performed under non-stress conditions during which the preference for YidC insertase may not be as critical. Membrane proteins that colocalized with S. mutans YidC1/YidC2 in the middle molecular weight region during formaldehyde cross-linking experiments were predominantly those with few TM domains, while YidC1-SecY/YajC and SRP-YidC2 pathway do not appear to distinguish single-pass and multipass membrane proteins. Different from the discovery-based approach described in the current study, E. coli pathways have largely been investigated in highly defined systems, whereby holotranslocon components were overexpressed and modified to track *in vivo* interactions by cross-linking assays or in which purified proteins were incorporated into artificial liposomes and studied using small membrane proteins as model substrates (16, 18, 61). A major finding of the current interactome analyses is that membrane protein translocation in S. mutans appears largely cotranslational, plausibly operating within a larger network of proteins that includes those involved in replication, transcription, and cell division. Numerous ribosomal proteins were associated with all three apparent S. mutans transport pathways consistent with the well-established cotranslational nature of membrane protein insertion. Taken together, the current results add to our understanding of the organization and respective substrates of distinct protein transport pathways in a Gram-positive bacterium. This information will facilitate future research regarding the underlying biology of a prevalent oral pathogen.

## MATERIALS AND METHODS

### Bacterial strains and growth conditions.

Bacterial strains, including S. mutans and E. coli, and plasmids used in this work are listed in Table S5 in the supplemental material. S. mutans cultures were routinely grown at 37°C in Todd-Hewitt broth (BBL, Becton Dickinson) supplemented with 0.3% yeast extract (THYE). Erythromycin (10 μg/ml) was added when appropriate.

10.1128/mSphere.01308-20.9 TABLE S5 Bacterial strains and plasmids. Download Table S5, PDF file, 0.2 MB. Copyright © 2021 Vasquez et al. 2021 Vasquez et al. https://creativecommons.org/licenses/by/4.0/ This content is distributed under the terms of the Creative Commons Attribution 4.0 International license.

All E. coli strains were routinely grown at 37°C or 30°C in Luria-Bertani (LB) broth or agar as mentioned in the text. Ampicillin (100 μg/ml) and/or kanamycin (50 μg/ml) was used for maintaining plasmids. LB agar reporter plates contained ampicillin (100 μg/ml), kanamycin (50 μg/ml), 5-bromo-4-chloro-3-indolyl-β-d-galactopyranoside (X-gal; 40 μg/ml) and isopropyl β-d-1-thiogalactopyranoside (IPTG; 0.5 mM).

### Formaldehyde cross-linking and Western blotting of whole-cell lysates.

Paraformaldehyde (Sigma-Aldrich) was added to 4% (wt/vol) in phosphate-buffered saline (PBS), pH 7.4, and stirred at 65°C with drop-by-drop addition of 1 M NaOH until dissolution was complete. The solution was cooled to room temperature (RT), adjusted to pH 7.4, filtered (0.22 μm), and stored at 4°C for up to 4 weeks. Fifty milliliters of cells from mid-log-phase S. mutans cultures (optical density at 600 nm [OD_600_] of ∼0.6) were harvested by centrifugation at 5,300 × *g* for 30 min at 4°C and washed twice with 10 ml PBS. The cell pellet was resuspended in 9.6 ml 0.4% formaldehyde solution and incubated for 15 min at 37°C with gentle shaking (Biometra OV5 3107A INC). The optimal concentration of 0.4% formaldehyde was established in pilot titration experiments. The reaction was quenched by the addition of 0.4 ml of 250 mM Tris (pH 7.4) (final concentration of 10 mM) and incubation at 37°C for 15 min. Paraformaldehyde-treated cells were pelleted by centrifugation, washed twice with PBS as described above, and resuspended to a final volume of 1 ml. Control cells were handled in the same way without formaldehyde. Whole-cell lysates were prepared from the cross-linked and untreated cell suspensions by glass bead breakage in a Mini-Bead Beater 8 apparatus (BioSpec Products, Inc., Bartlesville, OK) for four 40-s cycles with 1 min cooling on ice between each cycle. Cell lysate samples were electrophoresed on 4 to 20% precast gels (Bio-Rad Laboratories, Hercules, CA) in Tris-glycine-sodium dodecyl sulfate (SDS) buffer. Replicate gels were stained with Coomassie blue R 250 or transblotted onto Immobilon polyvinylidene difluoride (PVDF) membranes (Bio-Rad Laboratories, Hercules, CA), reacted with affinity-purified YidC1 or YidC2 C-terminus-specific polyclonal rabbit antibodies (1:1,000) (62) or anti-Ffh or anti-FtsY polyclonal rabbit antisera (1:1,000) (55), followed by horseradish peroxidase-labeled anti-rabbit IgG (MP Biomedicals, Irvine, CA) (1:5,000), and developed using the enhanced-chemiluminescence (ECL) Western blotting system (GE Healthcare).

### Coupling of anti-YidC2 antibodies to Dynabeads and immunocapture of protein complexes.

Five milligrams of M-280 tosylactivated Dynabeads (Invitrogen) was washed twice with 1 ml of 0.1 M Na-phosphate buffer (pH 7.4). The beads were coupled to affinity-purified rabbit polyclonal YidC2-specific antibodies generated against synthetic peptides corresponding to the C-terminal tail (NPPKPFKSNARKDITPQANNDKKLIT) and cytoplasmic loop 1 between TM2 and TM3 (SEKMAYLKPVFDPIQERMKNC). Beads were reacted at 37°C overnight with slow end-over-end rotation (Roto-Torque, Cole-Parmer, Chicago Illinois) in a final volume of 150 μl in 0.1 M Na-phosphate buffer (pH 7.4) containing 50 μg of each purified antibody preparation and 3 M ammonium sulfate. Following incubation, the tube was placed next to a magnet, and the supernatant was removed. Unbound antibodies were removed from the beads by washing first with 1 ml PBS containing 0.5% Triton X-100 and second with freshly made 0.5 N NH_4_OH, 0.5 mM EDTA, until the *A*_280_ of the wash supernatant was zero. Antibody (Ab)-coated beads were washed three times with 1 ml PBS, resuspended in 100 μl PBS, and reacted with ∼700 μl formaldehyde-cross-linked whole-cell lysate samples (∼8 mg/ml) derived from S. mutans strain NG8 (wild type) or PC398 (Δ*yidC2*) or with control samples prepared without formaldehyde for 3 h at 4°C with gentle end-over-end rotation. Next, beads were separated with a magnet and washed six times with 1 ml PBS. Ab-captured proteins were eluted with 0.5 ml freshly made 0.5 N NH_4_OH, 0.5 mM EDTA, and vortexing in an Eppendorf tube adapter (Vortex Mixer, Fisher Scientific) set at medium speed for 20 min at RT. Beads were removed with a magnet, and the eluate was snap-frozen in liquid nitrogen and dried overnight at RT in a SpeedVac vacuum concentrator (Savant, Famingdale, NY). Twenty microliters of SDS sample buffer (62.5 mM Tris [pH 6.8], 10% glycerol, 0.2% SDS, 0.02% bromophenol blue) were added to each dried sample and incubated for 10 min at 65°C. The samples were electrophoresed on 4 to 20% gradient SDS-polyacrylamide gels (Bio-Rad, Hercules, CA) and analyzed by Western blotting using anti-YidC2 C-terminus-specific antibodies as described above. Controls included non-cross-linked samples prepared without formaldehyde and a Western blot developed with horseradish peroxidase (HRP)-conjugated goat anti-rabbit secondary antibody only.

### Preparation of gel slices for protein identification by mass spectrometry.

SDS-polyacrylamide gels were rinsed in Optima liquid chromatography (LC)-mass spectrometry (MS) grade water (Fisher Scientific) three times, fixed for 15 min with 50% methanol and 7% acetic acid (Fisher Scientific), and stained with GelCode blue stain reagent (Thermo Scientific) according to the manufacturer’s instructions. Gel slices corresponding to gel-shifted regions identified by Western blotting with anti-YidC1, anti-YidC2, anti-Ffh, or anti-FtsY specific antibodies in the formaldehyde-cross-linked UA159 whole-cell lysate, but absent from the non-cross-linked control sample, were excised for *in situ* proteolysis. Similarly, a band detected by Western blotting with anti-YidC2 antibodies in the Dynabead eluate of the NG8 formaldehyde cross-linked sample, but not the Δ*yidC2* mutant strain or non-cross-linked control samples, was excised for proteolysis from the same location of SDS-polyacrylamide gels of all four samples. Gel slices were washed twice in nanopure water for 5 min and then destained with 1:1 (vol/vol) methanol−50 mM ammonium bicarbonate for 10 min with two changes. Gel slices were dehydrated with 1:1 (vol/vol) acetonitrile−50 mM ammonium bicarbonate, then rehydrated, and incubated with dithiothreitol (DTT) solution (25 mM in 100 mM ammonium bicarbonate) for 30 min prior to the addition of 55 mM iodoacetamide in 100 mM ammonium bicarbonate solution. Gel slices were incubated for an additional 30 min in the dark, then washed with two cycles of water, and dehydrated with 1:1 (vol/vol) acetonitrile and 50 mM ammonium bicarbonate. Protease was driven into the gel pieces by rehydrating them in 12 ng/ml trypsin in 0.01% ProteaseMAX surfactant (Promega) for 5 min. Gel pieces were next overlaid with 40 μl of 0.01% ProteaseMAX surfactant: 50 mM ammonium bicarbonate and gently mixed on an orbital shaker for 1 h. Digestion was stopped by the addition of 0.5% trifluoroacetic acid. MS analysis was performed immediately to ensure high-quality tryptic peptides with minimal nonspecific cleavage.

### Mass spectrometry analysis.

Nano-liquid chromatography tandem mass spectrometry (Nano-LC/MS/MS) was performed on a Thermo Scientific Q Exactive HF Orbitrap mass spectrometer equipped with an EASY Spray nanospray source (Thermo Scientific) operated in positive ion mode or on a quadrupole time of flight (Q-TOF) instrument. The LC system was an UltiMate 3000 RSLCnano system from Thermo Scientific. Mobile phase A was water containing 0.1% formic acid acetic acid, and mobile phase B was acetonitrile with 0.1% formic acid. Five microliters of each sample was first injected onto a Thermo Fisher Scientific Acclaim Trap Cartridge (C_18_ column, 75-μm inner diameter [ID], 2-cm length, 3-μm 100-Å pore size) and washed with mobile phase A to desalt and concentrate the peptides. The injector port was switched to inject, and the peptides were eluted off the trap onto the column. An EASY Spray PepMAP column from Thermo Scientific was used for chromatographic separations (C_18_, 75-μm ID, 25-cm length, 3-μm 100 Å pore size). The column temperature was maintained at 35°C as peptides were eluted directly off the column into the LTQ system using a gradient of 2 to 80% mobile phase B over 45 min, with a flow rate of 300 nl/min. The total run time was 60 min. The MS/MS was acquired according to standard conditions established in the lab. The EASY Spray source operated with a spray voltage of 1.5 kV and a capillary temperature of 200°C. The scan sequence of the mass spectrometer was based on the TopTen method; the analysis was programmed for a full scan recorded between 350 and 2,000 Da, and a MS/MS scan to generate product ion spectra to determine amino acid sequence in consecutive instrument scans of the 10 most abundant peaks in the spectrum. The AGC Target ion number was set at 30,000 ions for full scan and 10,000 ions for MSn mode. Maximum ion injection time was set at 20 ms for full scan and 300 ms for MSn mode. The micro scan number was set at 1 for both full scan and MSn scan. The collision-induced dissociation (CID) fragmentation energy was set at 35%. Dynamic exclusion was enabled with a repeat count of 1 within 10 s, a mass list size of 200, and an exclusion duration of 350 s. The low mass width was 0.5, and the high mass width was 1.5.

### Database searching.

All MS/MS samples were analyzed using Sequest (XCorr Only) (Thermo Fisher Scientific, San Jose, CA, USA; version IseNode in Proteome Discoverer 2.2.0.388 or Mascot Server 2.7). Sequest (XCorr Only) was set up to search Streptococcus mutans UA159 or NG8 (GenBank accession no. AE014133.2 or CP013237.1, respectively). Sequest (XCorr Only) was searched with a fragment ion mass tolerance of 0.020 Da and a parent ion tolerance of 10.0 ppm.

### Criteria for protein identification.

Scaffold (version Scaffold_4.8.6; Proteome Software Inc., Portland, OR) was used to validate MS/MS-based peptide and protein identifications. Peptide identifications were accepted if they could be established at greater than 95.0% probability by the Peptide Prophet algorithm (63) with Scaffold delta-mass correction. Protein identifications were accepted if they could be established at greater than 99.0% probability and contained at least one identified peptide. Protein probabilities were assigned by the Protein Prophet algorithm (64).

### Two-dimensional difference gel electrophoresis (DIGE) analysis of S. mutans proteins captured with GST-YidC1 compared to GST-YidC2 C-terminal tail fusion proteins.

The C-terminal fragment (bp 682 to 816) of *yidC1* was amplified by PCR using primers NL5F and NL5R (Table S6). The C-terminal fragment (bp 742 to 933) of *yidC2* was amplified by PCR using primers NL6F and NL6Rb (Table S6). S. mutans UA159 genomic DNA was used as the template. PCR products were digested with BamHI and SalI and ligated to corresponding restriction enzyme sites in the pGEX-4T-2 vector. The vector encoding only GST was transformed into BL21(DE3) (Thermo Fisher Scientific). Plasmids encoding GST-YidC1CT or GST-YidC2CT were transformed into BL21(DE3) Star (Thermo Fisher). GST-YidC1CT expression was induced with 0.5 mM isopropyl-β-d-thiogalactopyranoside (IPTG) for 6 h at 30°C. Expression of GST and GST-YidC2CT was induced with 1 mM IPTG for 4 h at 37°C. Cells were harvested by centrifugation at 11,325 × *g* for 15 min and resuspended in 25 ml PBS. Cell suspensions were supplemented with 1 mM phenylmethylsulfonyl fluoride (PMSF) (Acros Organics) and protease inhibitor cocktail (1 mini tablet/25 ml) (Roche Diagnostics GmbH). Cell lysis was performed using an Avestin EmulsiFlex-C5 high-pressure homogenizer (Avestin Inc., Ottawa, Ontario, Canada) at a pressure of 15,000 to 20,000 lb/in^2^ for three cycles. Cell debris was removed by centrifugation at 11,000 × *g* for 30 min, and the supernatants were filtered through a 0.22-μm syringe filter (Merck Millipore). Recombinant proteins were purified on an AKTA Purifier system (GE Healthcare) using a GSTrap column and elution with 50 mM Tris-HCl and 10 mM reduced glutathione (pH 8.0). Purified proteins were dialyzed in equilibration buffer (50 mM Tris, 150 mM NaCl [pH 8.0]) and incubated with Pierce glutathione spin columns (Thermo Scientific) at RT for 1 h with gentle rotation according to the manufacturer’s instructions. A fresh whole-cell lysate of S. mutans UA159 was filtered through a 0.22-μm syringe filter (Merck Millipore) and incubated with the GST and GST fusion protein-bound glutathione spin columns overnight at 4°C with gentle rotation. The column was washed four times with PBS, and bound proteins were eluted with 50 mM Tris and 150 mM NaCl (pH 8) containing 10 mM reduced glutathione. Eluates were separated by electrophoresis through 4 to 20% gradient SDS-polyacrylamide gels (Bio-Rad, Hercules, CA), visualized by Coomassie blue staining to confirm protein capture, and then sent to Applied Biomics (Hayward, CA) on dry ice for proteomic analysis. Proteins captured with GST, GST-YidC1CT, or GST-YidC2CT were labeled with CyDye DIGE blue Cy5, red Cy3, or green Cy2 fluors, respectively, separated on a single two-dimensional (2D) gel electrophoresis, and the gel was analyzed for spot picking, followed by trypsin digestion for mass spectrometry protein identification. Peptides were subjected to tandem matrix-assisted laser desorption ionization-time of flight (MALDI-TOF) for peptide mass fingerprinting and MALDI-TOF/TOF for identification of peptide sequences which were searched against S. mutans UA159 database from NCBI and SwissProt using MASCOT search engine (Matrix Science). Proteins with a protein score or total ion confidence interval (CI) greater than 95% were considered significant.

10.1128/mSphere.01308-20.10 TABLE S6 List of oligonucleotides. Download Table S6, PDF file, 0.5 MB. Copyright © 2021 Vasquez et al. 2021 Vasquez et al. https://creativecommons.org/licenses/by/4.0/ This content is distributed under the terms of the Creative Commons Attribution 4.0 International license.

### Bacterial two-hybrid assays.

Genes encoding selected proteins were PCR amplified using primers listed in Table S6. PCR products were cloned in pKT25 and pUT18C vectors as described before (54). In order to study protein-protein interaction, two recombinant plasmids expressing proteins as C-terminal fusions of T18 and T25 fragments were cotransformed into CaCl_2_ competent BTH101 cells and selected on LB plates containing ampicillin (100 μg/ml), kanamycin (50 μg/ml), and isopropyl β-d-1-thiogalactopyranoside (IPTG; 0.5 mM). Three different isolates were picked for each transformation experiment and grown in 1 ml LB supplemented with appropriate antibiotics and 0.5 mM IPTG overnight at 30°C with shaking. Four microliters of each culture were spotted on LB agar selection plates containing 0.5 mM IPTG and 40 μg/ml X-gal. Plates were incubated for 48 hours at 30°C before documentation. Data shown are representative of three independent biological experiments with three biological replicates each.

### Zone of inhibition assay.

Overnight grown cultures in THYE were diluted 1:50 and grown until mid-log phase (OD_600_ of 0.2). One hundred microliters of culture mixed with 4 ml of THYE top agar (THYE plus 0.7% agar) at 50°C and overlaid on a THYE agar plate. Sterile filter discs were placed in the center of the plate and spotted with 10 μl of 5,000-U/ml bacitracin stock solution. Plates were incubated for 24 h at 37°C in 5% CO_2_ atmosphere before documentation. Three independent experiments were performed in duplicate, and *P* values were calculated from Student *t* test.

### Bioinformatic analyses.

Amino acid sequences of proteins identified in all the experiments were downloaded from the S. mutans strain NG8 assembly database (CP013237.1) or UA159 assembly database (AE014133.2), and analyzed for the presence and number of transmembrane domains using the webtool TMHMM v2.0 (65). Functional analysis of proteins was conducted using the Database for Annotation, Visualization, and Integrated Discovery (DAVID) bioinformatics tool 6.8 (http://david.abcc.ncifcrf.gov/) (66). Protein-protein interaction (PPI) network analysis was performed using the STRING (Search Tool for the Retrieval of Interacting Genes/Proteins) database with a minimum required interaction score set to high confidence (0.700) (67). YidC1 and YidC2 were manually added to the respective analyses, as these proteins themselves were not the part of uploaded data sets.

### Expression and purification of recombinant proteins.

Recombinant E. coli bacteria were induced to produce 50S ribosomal protein L2 or trL2 with 0.05 mM IPTG at RT overnight. Bacterial cells were harvested by centrifugation at 11,325 × *g* for 15 min and resuspended in 25 ml of 50 mM sodium phosphate, 300 mM sodium chloride, 10 mM imidazole (pH 7.4) supplemented with 1 mM phenylmethylsulfonyl fluoride (PMSF) (Acros Organics) and protease inhibitor cocktail (1 mini tablet/25 ml) (Roche Diagnostics GmbH). Cell lysis was performed using an Avestin EmulsiFlex-C5 high-pressure homogenizer (Avestin Inc., Ottawa, Ontario, Canada) at a pressure of 15,000 to 20,000 lb/in^2^ for three cycles. Cell debris was removed by centrifugation, and the supernatant was filtered through a 0.22-μm syringe filter (Merck Millipore). Recombinant proteins were purified on an AKTA Purifier system (GE Healthcare) using a HiTrap Talon column and eluted with 50 mM sodium phosphate and 300 mM sodium chloride (pH 7.4) containing 150 mM imidazole.

10.1128/mSphere.01308-20.1 TEXT S1 Supplemental methods. Details of plasmid construction for purification of L2, trL2, and SecA, expression and purification of recombinant proteins, and enzyme-linked immunosorbent assay (ELISA). Download Text S1, PDF file, 0.4 MB. Copyright © 2021 Vasquez et al. 2021 Vasquez et al. https://creativecommons.org/licenses/by/4.0/ This content is distributed under the terms of the Creative Commons Attribution 4.0 International license.
