# Supplementary material for: Protein Interactomes of Streptococcus mutans YidC1 and YidC2 Membrane Protein Insertases Suggest SRP Pathway-Independent- and -Dependent Functions, Respectively
Source: mSphere. 2021 Mar 3;6(2):e01308-20. doi: 10.1128/mSphere.01308-20 (PMC8546722; doi:10.1128/mSphere.01308-20)
Supplement: TABLE S1 [file msphere.01308-20-st001.pdf]

Table S1

Accession UA159	Accession NG8	Description	Molecular Weight (kDa)	Theoretical pI	No. of TM domains
**Upper gel slice**					
SMU_1626	AMF84950.1	50S ribosomal protein L1	24.5	9.2	0*
SMU_1496	AMF85057.1	Galactose-6-phosphate isomerase. subunit LacA	15.6	6.55	0
SMU_1303c	AMF85207.1	Putative dipeptidase	53	5.11	0*
SMU_1276c	AMF85231.1	Putative septation ring formation regulator	66.5	4.72	1
SMU_1247	AMF85264.1	Putative enolase	46.8	4.78	0*
SMU_1200	AMF85306.1	Putative ribosomal protein S1; sequence specific DNA-binding protein	43.7	5.11	0*
SMU_1191	AMF85313.1	6-Phosphofructokinase	35.7	5.64	0*
SMU_1190	AMF85314.1	Pyruvate kinase	54.3	5.21	0*
SMU_1007	AMF85464.1	Putative ABC transporter, permease protein	75.8	9.67	10
SMU_960	AMF85507.1	50S ribosomal protein L7/L12	12.4	4.49	0*
SMU_913	AMF85547.1	Putative NADP-specific glutamate dehydrogenase	48.2	5.57	0*
SMU_872	AMF85578.1	Putative PTS system, fructose-specific enzyme IIABC component	68.2	5.66	10
SMU_822	AMF85624.1	DNA-dependent RNA polymerase sigma subunit; major sigma factor (sigma 70/42)	42.5	4.93	0*
SMU_754	AMF85684.1	HPr(serine) kinase/phosphatase	34.9	5.07	0*
SMU_714	AMF85715.1	Translation elongation factor EF-Tu	43.9	4.97	0*
SMU_668c	AMF85754.1	Ribonucleotide reductase, large subunit	81.4	5.68	0*
SMU_667	AMF85755.1	Putative ribonucleotide reductase, small subunit	36.8	4.54	0*

SMU_650	AMF85770.1	Putative alanyl-tRNA synthetase (alanine-tRNA ligase)	97.1	5.33	0*
SMU_611	AMF85803.1	Putative ATP-dependent RNA helicase, DEAD-box family	58.4	9.74	0*
SMU_591c	AMF85820.1	Hypothetical protein	42.6	9.95	1
SMU_589	AMF85821.1	Putative DNA-binding protein	9.7	9.82	0*
SMU_557	AMF85848.1	Putative cell division protein DivIVA	30.8	4.53	0*
SMU_552	AMF85852.1	Putative cell division protein FtsZ	45.7	4.54	0*
SMU_551	AMF85853.1	Cell division protein FtsA	49.1	5.12	0*
SMU_540	AMF85862.1	Peroxide resistance protein Dpr	19.6	4.86	0*
SMU_421	AMF85969.1	Translation initiation factor 2	101.5	8.97	0*
SMU_402	AMF85987.1	Pyruvate formate-lyase	87.6	5.44	0*
SMU_368c	AMF86014.1	Conserved hypothetical protein, ribonuclease J	61.3	6.37	0*
SMU_361	AMF86021.1	Phosphoglycerate kinase	42	5.39	0*
SMU_360	AMF86022.1	Extracellular glyceraldehyde-3-phosphate dehydrogenase	36	6.06	0*
SMU_359	AMF86023.1	Translation elongation factor G	76.6	4.91	0*
SMU_286	AMF86092.1	Putative ABC transporter, ATP-binding protein ComA	85	8.6	6
SMU_235	AMF86132.1	Conserved hypothetical protein, protease	33	5.74	1
SMU_234	AMF86133.1	Threonine dehydratase	45.5	5.17	0*
SMU_233	AMF86134.1	Ketol-acid reductoisomerase	37.3	5.14	0*
SMU_163c	AMF86165.1	Hypothetical protein, PadR family transcriptional regulator	21.2	9.52	0
SMU_155	AMF86173.1	Polyribonucleotide nucleotidyltransferase	80	5.26	0*
SMU_148	AMF86175.1	Putative alcohol-acetaldehyde dehydrogenase	96.9	5.72	0*

SMU_130	AMF86190.1	Putative dihydrolipoamide dehydrogenase	61.7	4.96	0*
SMU_99	AMF86207.1	Fructose-1.6-biphosphate aldolase	31.4	5.05	0*
SMU_91	AMF86211.1	Peptidyl-prolyl isomerase RopA (trigger factor)	47.5	4.55	0*
SMU_82	AMF86218.1	Heat shock protein. DnaK (HSP-70)	65.2	4.73	0*
SMU_15	AMF86268.1	Putative cell division protein FtsH	71.7	6.99	2
SMU_2157	AMF86288.1	Inosine monophosphate dehydrogenase	53.1	5.96	0*
SMU_2085	AMF86348.1	Recombination protein RecA	41.4	5.36	0*
SMU_2047	AMF86393.1	Putative PTS system, glucose-specific IIABC component	78.4	6.98	9
SMU_2038	AMF86400.1	Putative PTS system, trehalose-specific IIABC component	70.8	6.21	10
SMU_2032	AMF86405.1	30S ribosomal protein S2	29.1	5.12	0*
SMU_2031	AMF86406.1	Putative translation elongation factor TS	37.7	4.98	0*
SMU_2026c	AMF86411.1	30S ribosomal protein S10	4.1	9.54	0*
SMU_2167	AMF86415.1	50S Ribosomal Protein L2	30.2	10.64	0*
SMU_2021	AMF86418.1	30S ribosomal protein S3	23.2	9.58	0*
SMU_2009	AMF86429.1	30S ribosomal protein S5	17.1	10.05	0*
SMU_2002	AMF86437.1	30S ribosomal protein S11	13.4	11.43	0*
SMU_2001	AMF86438.1	DNA-directed RNA polymerase. alpha subunit	34.5	4.87	0*
SMU_1990	AMF86448.1	DNA-dependent RNA polymerase, beta subunit	132.6	5.19	0*
SMU_1989	AMF86449.1	DNA-dependent RNA polymerase, beta' subunit	136	6.8	0*

SMU_1960c	AMF86476.1	Putative PTS system, mannose-specific IIB component	18.2	8.03	0*
SMU_1954	AMF86481.1	Putative chaperonin GroEL	57.1	4.79	0*
SMU_1858	AMF86551.1	30S ribosomal protein S18	9.2	10.8	0*
SMU_1838	AMF86568.1	Preprotein translocase subunit SecA	95.4	5.25	0*
SMU_697	AMF86667.1	Putative translation initiation factor IF3	20.1	9.92	0*
SMU_2155	AMF86290.1	Conserved hypothetical protein, S4 RNA-binding domain-containing	13.8	9.92	0*
SMU_1115	AMF85378.1	Lactate dehydrogenase	35.2	5.01	0
SMU_1477	AMF85074.1	Putative tRNA isopentenylpyrophosphate transferase, tRNA dimethylallyltransferase	33.2	6.67	0*
**Middle gel slice**					
SMU_1687	AMF84897.1	Putative manganese-dependent inorganic pyrophosphatase	33.4	4.92	0*
SMU_1656	AMF84926.1	Putative phosphoserine aminotransferase	40.3	5.31	0*
SMU_1626	AMF84950.1	50S ribosomal protein L1	24.5	9.2	0*
SMU_1496	AMF85057.1	Galactose-6-phosphate isomerase, subunit LacA	15.6	6.55	0
SMU_1331c	AMF85187.1	Putative transposase	21.9	9.91	0
SMU_1203	AMF85304.1	Putative branched-chain amino acid aminotransferase IlvE	37.7	5.2	0*
SMU_629	AMF85790.1	Putative manganese-type superoxide dismutase, Fe/Mn-SOD	22.6	5.12	0
SMU_591c	AMF85820.1	Hypothetical protein	42.6	9.95	1
SMU_589	AMF85821.1	Putative DNA-binding protein	9.7	9.82	0*
SMU_500	AMF85905.1	Putative ribosome-associated protein	21.1	6.83	0*
SMU_364	AMF86018.1	Glutamine synthetase type 1; glutamate--ammonia ligase	49.9	5.25	0*

SMU_317	AMF86060.1	Putative tetrahydrodipicolinate succinylase	24.1	4.77	0*
SMU_163c	AMF86165.1	Hypothetical protein, PadR family transcriptional regulator	21.2	9.52	0
SMU_2164	AMF86282.1	Serine protease HtrA	43	8.41	1
SMU_2032	AMF86405.1	30S ribosomal protein S2	29.1	5.12	0*
SMU_2167	AMF86415.1	50S Ribosomal Protein L2	30.2	10.64	0*
SMU_2021	AMF86418.1	30S ribosomal protein S3	23.2	9.58	0*
SMU_2015	AMF86424.1	50S ribosomal protein L5	19.8	9.5	0*
SMU_2010	AMF86428.1	50S ribosomal protein L18	12.9	10.4	0*
SMU_2003	AMF86436.1	30S ribosomal protein S13	13.4	10.52	0*
SMU_1989	AMF86449.1	DNA-dependent RNA polymerase, beta' subunit	136	6.8	0*
SMU_1879	AMF86535.1	Putative PTS system, mannose-specific component IID	34.2	9.42	4
SMU_1790c	AMF86598.1	Putative transcriptional regulator	28	8.75	0
SMU_2127	AMF86313.1	Succinate-semialdehyde dehydrogenase	50.6	4.95	0*
SMU_1525	AMF85037.1	UDP-N-acetylglucosamine 1-carboxyvinyltransferase	45.6	5.64	0*
SMU_1693	AMF84891.1	Hemolysin	49.8	4.35	4
SMU_1591	AMF84979.1	Catabolite control protein A	36.6	6.41	0*
SMU_466	AMF85933.1	Aminopeptidase	50.6	5.38	0*
SMU_1146c	AMF85348.1	Two-component system response regulator, OmpR family	26.3	6.65	0
SMU_1820c	AMF86582.1	Glutamyl-tRNA amidotransferase	52.8	5.18	0
SMU_882	AMF85568.1	Sugar ABC transporter ATP-binding protein MsmK	42	5.92	0*

SMU_40	AMF86248.1	Hypothetical protein APQ13_07375 (ParE _toxin)	12.9	9.3	1
SMU_70	AMF86229.1	Threonine synthase	53.9	5.45	0*
SMU_1297	AMF85213.1	Phosphoesterase	34.6	6.08	0
SMU_1536	AMF85026	Putative starch (bacterial glycogen synthase)	54.4	4.99	0*
SMU_1731	AMF86638.1	Putative UDP-N-acetyl muramate-alanine ligase	50.8	5.65	0*
SMU_694c	AMF85731.1	Putative ferrodoxin (4Fe-4S)	71.8	5.03	0
SMU_1812	None	Putative transposase, ISSmu2	49.8	9.93	0
**Lower gel slice**					
SMU_667	AMF85755.1	Putative ribonucleotide reductase, small subunit	36.8	4.54	0*
SMU_1447c	AMF85098.1	Conserved hypothetical protein peptide ABC transporter substrate-binding protein	35.9	9.5	1
SMU_1617	AMF84958.1	GTP-binding protein; Era-like protein	34.2	8.21	0*
SMU_1620	AMF84956.1	Putative phosphate starvation-induced protein PhoH	35.7	5.78	0*
SMU_1430	AMF85112.1	Putative cobyric acid synthase CobQ, glutamine amidotransferase	29.6	5.49	0*
SMU_696	AMF85729.1	putative cytidylate kinase	25.2	8.22	0*
SMU_1040c	AMF85443.1	Putative oxidoreductase, short-chain dehydrogenase/reductase, NAD(P)-dependent oxidoreductase	27.6	9.01	0*
SMU_187c	AMF86145.1	Conserved hypothetical protein, nitrogen fixation protein NifR	35.6	6.46	0*
SMU_754	AMF85684.1	HPr(serine) kinase/phosphatase	34.9	5.07	0*
SMU_1054	AMF85430.1	Putative glutamine amidotransferase	26.1	4.72	0*

SMU_1417c	AMF85125.1	Putative oleoyl-acyl carrier protein thioesterase	28.7	6.05	0*
SMU_1203	AMF85304.1	Putative branched-chain amino acid aminotransferase IlvE	37.7	5.2	0*
SMU_1144	AMF85350.1	Putative tRNA pseudouridine 5S synthase	32.9	7.36	0*
SMU_1595	AMF84977.1	Putative carbonic anhydrase precursor	29.2	9.39	1
SMU_1225	AMF85285.1	Putative transcriptional regulator, LysR family transcriptional regulator	34.9	5.53	0*
SMU_623c	AMF85796.1	Putative deacetylase	34.7	9.92	1
SMU_349	AMF86033.1	Dimethyladenosine transferase, 16S rRNA methyltransferase	32.8	5.78	0*
SMU_506	AMF85899.1	Putative type II restriction endonuclease	36.4	6.83	0*
SMU_834	AMF85612.1	Conserved hypothetical protein, glycosyl transferase	35.8	6.58	2
SMU_1134c	AMF85360.1	Putative phosphate ABC transporter, ATP-binding protein	28	8.03	0*
SMU_406c	AMF86674.1	Conserved hypothetical protein, HAD family hydrolase	31.1	9.26	0*
SMU_415	AMF85975.1	Conserved hypothetical protein, aminoglycoside phosphotransferase	30.4	9.09	0*
SMU_504	AMF85901.1	Putative site-specific DNA-methyltransferase	33.5	6.83	0*
SMU_1717c	AMF84869.1	Conserved hypothetical protein, non-canonical purine NTP pyrophosphatase	36.4	5.15	0*
SMU_2047	AMF86393.1	Putative PTS system. glucose-specific IIABC component	78.4	6.98	9
SMU_832	AMF85614.1	Hypothetical protein	50.6	9.58	11
SMU_743	AMF85693.1	Conserved hypothetical protein. haloacid dehalogenase	30.4	4.93	0*

SMU_933	AMF85531.1	Putative amino acid ABC transporter, periplasmic amino acid-binding protein	31.8	9.39	1
SMU_84	AMF86217.1	Putative tRNA pseudouridine synthase A	28.2	9.25	0*
SMU_1645	AMF84935.1	Putative tellurite resistance protein	33.8	7.18	0*
SMU_1307c	AMF85204.1	Conserved hypothetical protein, cyclic nucleotide-binding protein	29.1	5.96	2
SMU_20	AMF86266.1	Putative cell shape-determining protein MreC	29.6	9.09	1
SMU_2149c	AMF86296.1	Putative ABC transporter, ATP-binding protein; possible cobalt transport system	30.9	5.21	0*
SMU_1763c		Conserved hypothetical protein	28.7	5.54	0
SMU_135	AMF86185.1	Putative transcriptional regulator LysR family transcriptional regulator	34.4	9.01	0*
SMU_338	AMF86041.1	Putative RNA-binding protein, Jag family	36	7.46	0*
SMU_1979c	AMF86458.1	Conserved hypothetical protein, adenine methyltransferase	36.3	5.55	0
SMU_833	AMF85613.1	Putative glycosyltransferase	35	8.87	2
SMU_1732c	AMF86637.1	Conserved hypothetical protein	23.1	8.57	0*
SMU_1590	AMF84980.1	intracellular alpha-amylase	56.4	4.79	0*
SMU_1427c	AMF85115.1	Conserved hypothetical protein	35.3	8.68	1
SMU_1566	AMF85000.1	Putative maltose operon transcriptional repressor, LacI family transcriptional regulator	38.1	9.28	0*
SMU_728	AMF85704.1	Putative oxidoreductase	32.2	6.42	0*
SMU_1398	AMF85139.1	Putative transcriptional regulator	32.2	6.74	0*

SMU_1046c	AMF85437.1	Putative GTP pyrophosphokinase	26.3	6.98	0
SMU_91	AMF86211.1	Peptidyl-prolyl isomerase RopA (trigger factor)	47.5	4.55	0*
SMU_118c	AMF86200.1	Putative esterase, S-formylglutathione hydrolase	31.4	6.37	0*
SMU_74	AMF86225.1	Conserved hypothetical protein, phosphoglycerate mutase	26.5	5.15	0*
SMU_1936c	AMF86497.1	Conserved hypothetical protein, DNA-directed RNA polymerase subunit delta	31.5	5.34	0*
SMU_937	AMF85527.1	Putative mevalonate diphosphate decarboxylase	34.9	8.43	0*
SMU_1465c	AMF85084.1	conserved hypothetical protein; replication protein DnaD-like	26.3	4.81	0*
SMU_1650	AMF84932.1	Putative endonuclease III (DNA repair)	23.3	8.6	0*
SMU_456	AMF85943.1	Putative undecaprenyl-phosphate-UDP-MurNAc-pentapeptide transferase	37.7	9.47	10
SMU_1200	AMF85306.1	putative ribosomal protein S1; sequence specific DNA-binding protein	43.7	5.11	0*
SMU_1831	AMF86574.1	Putative L-asparaginase	34.6	6.38	0*
SMU_341	AMF86677.1	Putative deoxyribonuclease, hydrolase TatD	34	5.91	0*
SMU_1786	AMF86602.1	Putative undecaprenyl pyrophosphate synthetase	28.5	8.92	0*
SMU_449	AMF85948.1	Putative gamma-glutamyl kinase	29.7	5.38	0*
SMU_1279c	AMF85228.1	Putative cell division protein RodA	45.3	8.97	10
SMU_852	AMF85595.1	Putative transcriptional regulator; CpsY-like	34.5	5.74	0*

		protein, LysR family transcriptional regulator			
SMU_1690	AMF84894.1	Integral membrane protein possibly involved in D-alanine export, D-alanyl-lipoteichoic acid biosynthesis protein DltB	49.9	9.66	10
SMU_257	AMF86113.1	Putative transmembrane protein, permease OppC	36.9	9.13	5
SMU_61	AMF86235.1	Putative transcriptional regulator, Cro/Cl family transcriptional regulator	35.2	5.22	0*
SMU_275	AMF86098.1	Putative L-ribulose 5-phosphate 4-epimerase	26.1	5.95	0
SMU_1111c	AMF85381.1	Conserved hypothetical protein	30.5	7.36	1
SMU_1445c	AMF85100.1	Putative ABC transporter, ATP-binding protein	28	5.97	0*
SMU_1140c	AMF85354.1	Conserved hypothetical protein inositol monophosphatase	25.7	5.48	0*
SMU_1695	AMF84889.1	Putative ABC transporter, ATP-binding protein; possible molybdenum transport system	29.3	9.17	0*
SMU_173	AMF86156.1	Putative ppGpp-regulated growth inhibitor	12.6	6.55	0
SMU_1867c	AMF86545.1	Putative alcohol dehydrogenase, Zn-dependent alcohol dehydrogenase	37.1	5.68	0*
SMU_1578	AMF84991.1	Putative biotin operon repressor	34.5	5.99	0*
SMU_826	AMF85620.1	Rhamnosyltransferase	36.6	8.72	0*
SMU_1450	AMF85096.1	Putative amino acid permease	52.1	9.77	12
SMU_503c	AMF85902.1	Hypothetical protein	22.5	9.31	1
SMU_1249c	AMF85263.1	Hypothetical protein	24.5	9.45	1
SMU_1305c	AMF85206.1	Conserved hypothetical protein	35.7	5.21	0*

SMU_2006	AMF86432.1	Putative preprotein translocase SecY protein	47.8	9.57	9
SMU_1669	AMF84913.1	Putative ABC transporter, branched chain amino acid-binding protein	41.2	9.01	0*
SMU_1175	AMF85327.1	Putative sodium/amino acid (alanine) symporter	48.3	9.42	11
SMU_246	AMF86122.1	Putative glycosyl transferase N-acetylglucosaminyltransferase), RgpG	42.6	9.7	11
SMU_1954	AMF86481.1	Putative chaperonin GroEL	57.1	4.79	0*
SMU_742	AMF85694.1	Conserved hypothetical protein, HAD family hydrolase	31.4	5.21	0*
SMU_714	AMF85715.1	Translation elongation factor EF-Tu	43.9	4.97	0*
SMU_507	AMF85897.1	Putative transcriptional regulator (DeoR family), cytochrome C	28.4	8.22	0
SMU_1661c	AMF84921.1	Putative signal peptidase II	30.4	5.47	0*
SMU_1050	AMF85434.1	Putative phosphoribosylpyrophosphate synthetase, PRPP synthetase	35.6	5.43	0*
SMU_1787c	AMF86601.1	Putative secreted protein, preprotein translocase subunit YajC	14.1	4.49	1
SMU_1927	AMF86505.1	Putative ABC transporter, ATP-binding protein	26.3	5.77	0*
SMU_707c	AMF85720.1	Putative endolysin, N-acetylmuramoyl-L-alanine amidase	31.5	9.23	1
SMU_858	AMF85589.1	Putative aspartate transcarbamoylase	34.7	6.87	0*
SMU_1662	AMF84920.1	Putative DNA polymerase III, delta subunit	33.6	5.33	0*
SMU_770c	AMF85673.1	Putative manganese transporter	48.8	9.7	11

SMU_2155	AMF86290.1	Conserved hypothetical protein	13.8	9.92	0*
SMU_1126	AMF85367.1	Putative pantothenate kinase	35.7	6.57	0*
SMU_1659c	AMF84923.1	Conserved hypothetical protein,16S rRNA methyltransferase	32.7	6.05	0
SMU_356	AMF86026.1	Purine operon repressor	29.4	9.01	0*
SMU_837	AMF85609.1	Putative reductase, 2.5-diketo-D-gluconic acid reductase	31.6	5.54	0*
SMU_1084	AMF86658.1	Putative protoporphyrinogen oxidase, protein-(glutamine-N5) methyltransferase. release factor-specific	31.5	4.74	0*
SMU_484	AMF85919.1	Putative serine/threonine protein kinase	66.9	8.97	1
SMU_1994	AMF86444.1	Putative ABC transporter, ATP-binding protein. zinc ABC transporter ATP-binding protein	26.8	7.31	0*
SMU_15	AMF86268.1	Putative cell division protein FtsH	71.7	6.99	2
SMU_1673	AMF84909.1	Uracil phosphoribosyltransferase	23	6.7	0*
SMU_1161c		Hypothetical protein	28.6	5.47	1
SMU_1344c	AMF85176.1	Putative malonyl-CoA acyl-carrier-protein transacylase	47.1	6.8	0*
SMU_1493	AMF85060.1	Tagatose-1,6-bisphosphate aldolase	36.5	5.19	0*
SMU_1529	AMF85033.1	FoF1 membrane-bound proton-translocating ATPase, gamma subunit	32.3	5.57	0*
SMU_2158c	AMF86287.1	Putative tryptophanyl-tRNA synthetase	38.3	6.38	0*
SMU_252	AMF86116.1	Hypothetical protein	29.3	9.51	0*
SMU_745	AMF85691.1	Putative drug-export protein; multidrug resistance protein, XRE	50	9.91	14

		family transcriptional regulator			
SMU_322c	AMF86056.1	Glucose-1-phosphate uridylyltransferase	33.9	5.6	0*
SMU_1761c		Conserved hypothetical protein	55.3	7.96	0
SMU_1780	AMF86608.1	Conserved hypothetical protein recombination regulator RecX	30.7	8.72	0
SMU_1790c	AMF86598.1	Putative transcriptional regulator, MerR family transcriptional regulator	28	8.75	0
SMU_262	AMF86108.1	Putative ornithine carbamoyltransferase	38.5	5.15	0
SMU_172	AMF86157.1	Conserved hypothetical protein cell division protein FtsW	9.2	4.65	0
SMU_998	AMF85473.1	Putative ABC transporter, periplasmic ferrichrome-binding protein	37.7	9.74	1
SMU_987	AMF85485.1	Cell wall-associated protein precursor WapA	48.9	6.07	2
SMU_2159	AMF86286.1	Putative ABC transporter, ATP-binding protein	60.7	4.83	0*
SMU_678	AMF85744.1	Putative oxidoreductase, aldo/keto reductase family	31.5	5.11	0*

* Proteins identified as membrane-associated by Mishra et al. 2019 Molecular Oral Microbiology
