# Supplementary material for: Protein Interactomes of Streptococcus mutans YidC1 and YidC2 Membrane Protein Insertases Suggest SRP Pathway-Independent- and -Dependent Functions, Respectively
Source: mSphere. 2021 Mar 3;6(2):e01308-20. doi: 10.1128/mSphere.01308-20 (PMC8546722; doi:10.1128/mSphere.01308-20)
Supplement: TABLE S2 [file msphere.01308-20-st002.pdf]

Table S2.

Proteins identified in formaldehyde cross-linked WT, but not *ΔyidC2* cross-linked sample					
**Accession UA159**	**Accession NG8**	**Description**	**Molecular Weight (kDa)**	**Theoretical pI**	**No. of TM domains**
SMU_1571	AMF84996.1	Sugar ABC transporter ATP-binding protein	42.3	6.33	0*
SMU_597	AMF85815.1	Penicillin-binding protein Pbp2b	75	9.01	1
SMU_1784c	AMF86604.1	Metalloprotease RseP†	45.8	9.51	4
SMU_1989	AMF86449.1	DNA-directed RNA polymerase subunit beta'	134.9	6.54	0*
SMU_73	AMF86226.1	Hypothetical protein APQ13_07260	46.3	5.34	0*
SMU_973	AMF85495.1	Spermidine/putrescine ABC transporter ATP-binding protein	43.5	4.87	0*
SMU_717	AMF85712.1	UDP-N-acetylmuramoylpentapeptide-lysine N(6)-alanyltransferase	46.6	9.25	0*
SMU_137	AMF86183.1	NAD-dependent malic enzyme	59.6	4.87	0*
SMU_716	AMF85713.1	Peptidoglycan branched peptide synthesis protein†	47.7	9.04	0*
SMU_1512	AMF85049.1	Phenylalanine--tRNA ligase subunit alpha	39.2	5.99	0*
SMU_1276c	AMF85231.1	Septation ring formation regulator EzrA	66.6	4.72	1
SMU_1572	AMF84995.1	UDP-N-acetylglucosamine 1-carboxyvinyltransferase†	44.6	6.05	0*
SMU_300	AMF86073.1	Queuine tRNA-ribosyltransferase	43.2	6.61	0*
SMU_258	AMF86112.1	Peptide ABC transporter ATP-binding protein	39.1	5.91	0*
SMU_1838	AMF86568.1	Preprotein translocase subunit SecA	95.4	5.25	0*
SMU_533	AMF85869.1	Anthranilate synthase subunit II	20.8	5.12	0*
SMU_956	AMF85509.1	ATP-dependent Clp protease ATP-binding subunit	77.1	5.11	0*

SMU_1692	AMF84892.1	Pyruvate formate lyase-activating enzyme 1	30.1	7.8	0*
SMU_1930	AMF86502.1	Cytoplasmic membrane protein, LemA	21	5.72	1
SMU_1688	AMF84896.1	D-alanyl-lipoteichoic acid biosynthesis protein DltD	48.6	9.91	1
SMU_1942c	AMF86491.1	Amino acid ABC transporter substrate-binding protein	30.2	9.41	0*
SMU_484	AMF85919.1	Serine/threonine protein kinase	66.9	8.97	1
SMU_1321c	AMF85194.1	Carboxylate--amine ligase	45.7	5.53	0*
SMU_129	AMF86191.1	Iron ABC transporter ATP-binding protein	48.7	5.86	0
SMU_1787c	AMF86601.1	Preprotein translocase subunit YajC	14.1	4.49	1
SMU_127	AMF86193.1	Pyruvate dehydrogenase†	35.2	5.19	0*
SMU_1653	AMF84929.1	Phosphoglycerate dehydrogenase	42.8	6.2	0*
SMU_1879	AMF86535.1	PTS mannose family transporter subunit IID	34.2	9.42	4
	AMF86619.1	Deoxyguanosinetriphos phate triphosphohydrolase	53	6.96	0
SMU_428	AMF85963.1	Haloacid dehalogenase	30.1	5	0*
SMU_1719c	AMF84867.1	Hypothetical protein APQ13_00045	9.3	10.2	1
SMU_67	AMF86230.1	Acyltransferase	67.6	7.44	11
SMU_396	AMF85991.1	ABC transporter permease	30.7	8.98	5
SMU_59	AMF86237.1	Adenylosuccinate lyase	49.3	5.67	0*
SMU_1206c	AMF85301.1	GTP pyrophosphokinase	22.1	7.62	0*
SMU_440	AMF85955.1	Polyketide cyclase	15.7	4.55	0
SMU_871	AMF85579.1	1-Phosphofructokinase †	32.8	5.05	0*
	AMF86398.1	Hypothetical protein APQ13_08305	46.7	7.59	0
SMU_327	AMF86052.1	DNA repair protein RadA	51.1	7.23	0*
SMU_1663	AMF84919.1	Thymidylate kinase	23.9	5.74	0*
SMU_1194	AMF85310.1	ABC transporter	26.4	6.42	0*

SMU_1021	AMF85451.1	Citrate lyase subunit alpha†	55.4	5.57	0*
SMU_333	AMF86046.1	Hypothetical protein APQ13_06235	45.1	4.92	3
SMU_1922	AMF86510.1	Helicase loader	45.3	7.24	0*
SMU_1053	AMF85431.1	Rex family transcriptional regulator	23.8	8.53	0*
SMU_2017	AMF86422.1	50S ribosomal protein L14	13	10.27	0*
SMU_712	AMF85717.1	Phosphoenolpyruvate carboxylase	103.7	5.35	0*
SMU_546	AMF85858.1	GTP-binding protein TypA	68.4	5.07	0*
SMU_675	AMF85747.1	Phosphoenolpyruvate-protein phosphotransferase	63.3	4.68	0*
SMU_672	AMF85750.1	Isocitrate dehydrogenase	43.1	6.57	0*
SMU_116	AMF86202.1	Tagatose-bisphosphate aldolase	36.5	4.87	0
SMU_1482c	AMF85071.1	Hydrolase	23.2	7.11	0*
SMU_66	AMF86231.1	Hypothetical protein APQ13_07285	14.1	9.86	1
SMU_2150c	AMF86295.1	Energy-coupling factor transporter ATPase	31.6	5.27	0*
SMU_1877	AMF86537.1	PTS mannose transporter subunit IIAB	35.5	5.29	0*
SMU_1383	AMF85153.1	3-isopropylmalate dehydrogenase	36.7	5.44	0*
SMU_2072c	AMF86360.1	GNAT family acetyltransferase	21.1	6.68	0*
SMU_458	AMF85941.1	DEAD/DEAH box helicase	51.2	9.77	0*
SMU_1878	AMF86536.1	PTS mannose transporter subunit IIC	28.1	6.05	8
Not	AMF85254.1	Deoxyribonuclease HsdR	117.5	5.62	0
SMU_1124	AMF85369.1	Thymidine phosphorylase	46.5	6.46	0*
SMU_1002	AMF85469.1	DNA topoisomerase I	80.2	9.31	0*
SMU_1693	AMF84891.1	Hemolysin	49.8	4.48	4
SMU_1785	AMF86603.1	phosphatidate cytidylyltransferase	29	8.38	9
SMU_1488c	AMF85065.1	Hypothetical protein APQ13_01075	10.4	6.77	0

SMU_575c	AMF85832.1	Murein hydrolase transporter LrgA	17	9.63	4
SMU_1277	AMF85230.1	DNA topoisomerase IV subunit B	72.4	5.6	0*
SMU_1623c	AMF84953.1	RNA-binding protein	32.3	8.82	0*
**Proteins identified in non-cross-linked WT, but not *ΔyidC2* non-cross-linked sample**					
SMU_1493	AMF85060.1	Tagatose-bisphosphate aldolase	36.5	5.19	0*
SMU_962	AMF85505.1	Dehydrogenase	39.4	5.26	0*
SMU_871	AMF85579.1	1-phosphofructokinase†	32.8	5.05	0*
SMU_127	AMF86193.1	Pyruvate dehydrogenase†	35.2	5.19	0*
SMU_1298	AMF85212.1	50S ribosomal protein L31 type B	9.3	8.97	0*
SMU_2020	AMF86419.1	50S ribosomal protein L16	15.5	10.65	0*
SMU_1572	AMF84995.1	UDP-N-acetylglucosamine 1-carboxyvinyltransferase†	44.6	6.05	0*
SMU_2141	AMF86302.1	tRNA uridine 5-carboxymethylaminomethyl modification protein	70.4	7.31	0*
SMU_1495	AMF85058.1	Galactose-6-phosphate isomerase	18.9	6.21	0
SMU_716	AMF85713.1	Peptidoglycan branched peptide synthesis protein†	47.7	9.04	0*
SMU_1784c	AMF86604.1	Metalloprotease RseP†	45.8	9.51	4
SMU_1021	AMF85451.1	Citrate lyase subunit alpha†	55.4	5.57	0*
SMU_1012c	AMF85459.1	GntR family transcriptional regulator	26.6	6.95	0*
SMU_829	AMF85617.1	Glycosyl transferase	54.8	5.63	0*
SMU_1735	AMF86634.1	Acetyl-CoA carboxylase carboxyl transferase subunit beta	31.9	8.9	0*
SMU_1418	AMF85124.1	Coproporphyrinogen III oxidase	43.4	5.85	0
	AMF86085.1	Glycosidase	47.6	9.54	10
SMU_1856c	AMF86552.1	Hypothetical protein APQ13_09110	27.8	9.06	7
SMU_1279c	AMF85228.1	Rod shape-determining protein RodA	45.3	8.97	10

not present	AMF86238.1	Hypothetical protein APQ13_07320	33.2	5.12	1
SMU_554	AMF85850.1	Cell division protein SepF	21.6	5.9	0*
SMU_1516	AMF85045.1	PAS domain-containing sensor histidine kinase	51.7	4.89	1
SMU_1564	AMF86646.1	Maltose phosphorylase	86.7	5.36	0*
SMU_596	AMF85816.1	Phosphoglycerate mutase	26	5.29	0*
SMU_1937	AMF86496.1	Hydrolase	29.1	6.89	0*
SMU_30	AMF86256.1	Phosphoribosylformylglycinamidine synthase	135.1	4.98	0*
SMU_01	AMF86280.1	Chromosomal replication initiator DnaA	51.3	5.72	0*
SMU_1145c	AMF85349.1	Histidine kinase	50	9.25	2
SMU_383c	AMF86002.1	Oxidoreductase	39.5	8.87	0*
SMU_1419	AMF85123.1	TetR family transcriptional regulator	23	9.04	0*
SMU_883	AMF85567.1	Glucohydrolase	61.9	5.27	0*

† Indicates proteins found in both formaldehyde-cross-linked and non-cross-linked samples of the WT but not *ΔyidC2* mutant.

* previously identified as membrane-associated (Mishra et al., 2019)
