# Supplementary figures and images for: Protein Interactomes of Streptococcus mutans YidC1 and YidC2 Membrane Protein Insertases Suggest SRP Pathway-Independent- and -Dependent Functions, Respectively
Source: mSphere. 2021 Mar 3;6(2):e01308-20. doi: 10.1128/mSphere.01308-20 (PMC8546722; doi:10.1128/mSphere.01308-20)

**Fig. S1**

**A**

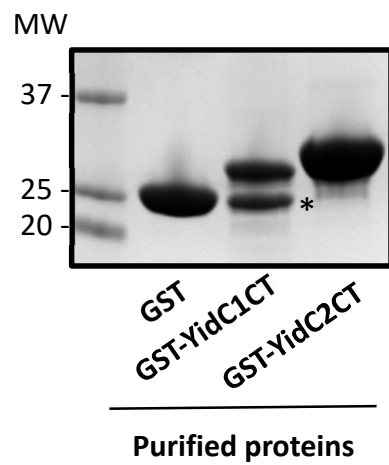

**B**

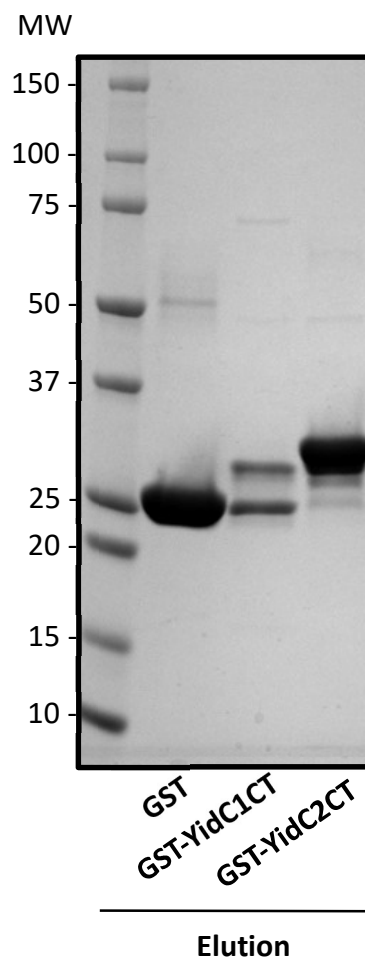

Supplement: FIG S1 [file msphere.01308-20-sf001.pdf]

Fig. S2

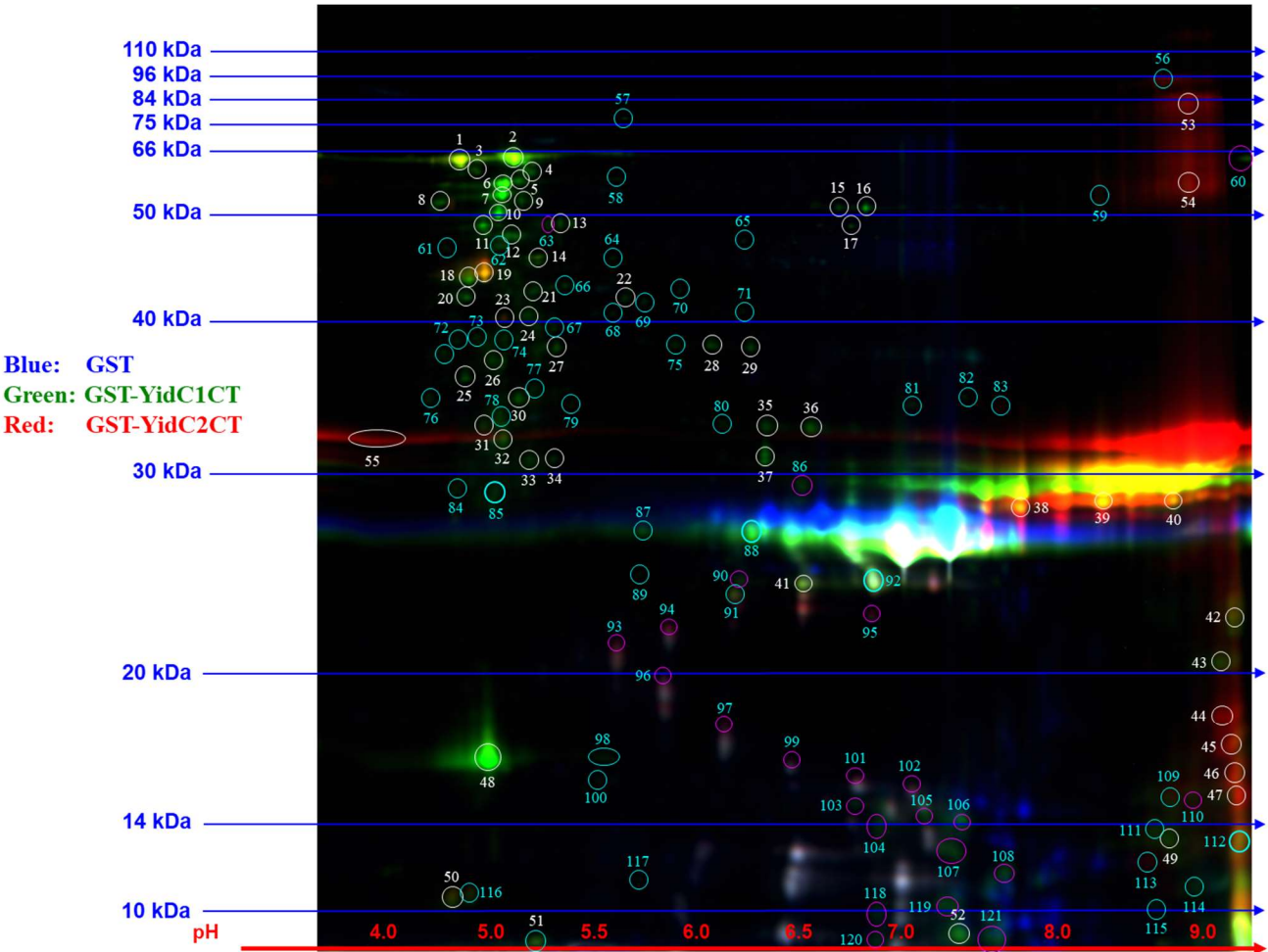

Supplement: FIG S2 [file msphere.01308-20-sf002.pdf]

**Fig. S3**

**A**

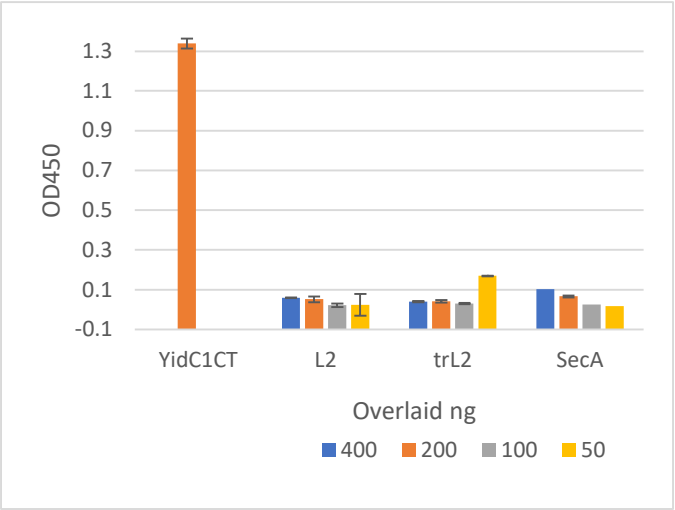

**B**

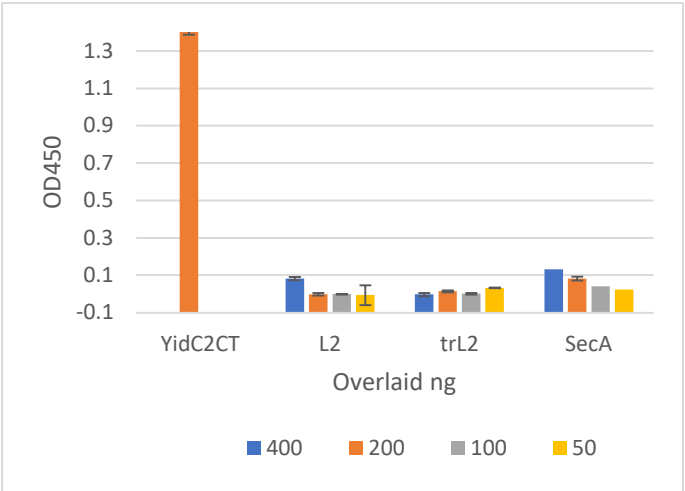

Supplement: FIG S3 [file msphere.01308-20-sf003.pdf]
