# Supplementary material for: Protein Interactomes of Streptococcus mutans YidC1 and YidC2 Membrane Protein Insertases Suggest SRP Pathway-Independent- and -Dependent Functions, Respectively
Source: mSphere. 2021 Mar 3;6(2):e01308-20. doi: 10.1128/mSphere.01308-20 (PMC8546722; doi:10.1128/mSphere.01308-20)
Supplement: TABLE S4 [file msphere.01308-20-st004.pdf]

Table S4:

YidC1CT							
**Accession UA159**	**Accession NG8**	**Protein name**	**No. of Spots**		**Molecular Weight (kDa)**	**Theoretical pI**	**No. of TM domains**
			**Green**	**Yellow**			
SMU_1581	AMF84989.1	DNA polymerase III, gamma/tau subunit	29	3	62.15	5.10	0*
SMU_15	AMF84978.1	Cell division protein FtsH	14		71.67	6.54	2
SMU_1216c	AMF85294.1	Putative amino acid ABC transporter, permease protein	10	1	24.29	8.76	6
SMU_2167	AMF86415.1	50S Ribosomal Protein L2	7	2	30.19	10.64	0*
SMU_1247	AMF85264.1	Enolase	6	1	46.82	4.67	0*
SMU_906	AMF85551.1	ABC transporter ATP-binding protein	5	2	66.82	6.44	5
SMU_486	AMF85917.1	Histidine kinase	4	2	38.09	5.80	2
SMU_1601	AMF84971.1	6-phospho-beta-D-glucosidase	1	4	55.37	5.17	0*
SMU_1357	None	Putative transposase fragment	2	3	9.50	5.26	0
SMU_1417c	AMF85125.1	Putative oleoyl-acyl carrier protein thioesterase	3	2	28.72	5.70	0*
SMU_82	AMF86218.1	Molecular chaperone DnaK	3	1	65.24	4.58	0*
SMU_2032	AMF86405.1	30S ribosomal protein S2	4		29.07	5.00	0*
SMU_2047	AMF86393.1	Putative PTS system, glucose-specific IIABC	4		78.36	6.47	10

SMU_714	AMF85715.1	Elongation factor Tu	3		43.89	4.84	0*
SMU_1190	AMF85314.1	Pyruvate kinase	2	1	54.33	5.09	0*
SMU_1599	AMF84973.1	Transcriptional regulator	2	1	76.69	5.89	0*
SMU_260	AMF86110.1	Nitroreductase family protein		2	22.37	4.95	0*
SMU_1704	AMF84882.1	DNA-binding transcriptional regulator, PadR family	2		12.64	9.17	0*
SMU_2080	AMF86352.1	LytTR family transcriptional regulator	2		17.17	9.96	0
SMU_123	AMF86196.1	DNA polymerase III PolC	1	1	165.46	5.11	0*
SMU_360	AMF86022.1	Type I glyceraldehyde-3-phosphate dehydrogenase	2		36.04	5.71	0*
SMU_233	AMF86134.1	Ketol-acid reductoisomerase	2		37.26	5.01	0*
SMU_2011	AMF86427.1	50S ribosomal protein L6	1	1	19.42	9.70	0*
SMU_355	AMF86027.1	CMP-binding factor	1	1	37.07	5.45	0*
SMU_651c	AMF85769.1	Putative ABC transporter, substrate-binding protein	1	1	36.69	9.17	1
SMU_1200	AMF85306.1	Putative ribosomal protein S1	1		43.66	5.00	0*
SMU_2135c	AMF86307.1	30S ribosomal protein S4	1		23.02	10.06	0*
SMU_1342	AMF85178.1	Non-ribosomal peptide synthetase		1	313.43	5.28	0*

SMU_169	AMF86159.1	50S ribosomal protein L13		1	16.20	9.87	0*
SMU_542	AMF85860.1	Glucose kinase	1		33.61	4.84	0*
SMU_99	AMF86207.1	Fructose-bisphosphate aldolase	1		31.40	4.96	0*
SMU_2001	AMF86438.1	DNA-directed RNA polymerase subunit alpha		1	34.54	4.76	0*
SMU_358	AMF86024.1	30S ribosomal protein S7		1	17.79	10.39	0*
SMU_515	AMF85885.1	Oleate hydratase		1	67.70	6.11	0
SMU_2012	AMF86426.1	30S ribosomal protein S8	1		14.68	9.10	0*
SMU_1530	AMF85032.1	ATP synthase F0F1 subunit alpha	1		54.33	4.85	0*
SMU_2018	AMF86421.1	30S ribosomal protein S17		1	10.01	10.18	0*
SMU_361	AMF86021.1	Phosphoglycerate kinase	1		42.01	5.23	0*
SMU_2042	AMF86396.1	Dextranase		1	94.42	4.99	1
SMU_297	AMF86076.1	DNA polymerase I (POL I)	1		99.39	5.23	0*
SMU_1461	AMF85088.1	Glucose-1-phosphate thymidylyltransferase	1		32.27	4.90	0*
SMU_365	AMF86017.1	Glutamate synthase	1		166.52	5.57	0*
SMU_585	AMF85825.1	DNA repair protein RecN	1		62.7	5.24	0*
SMU_675	AMF85747.1	PTS system transporter protein EI	1		63.35	4.57	0*
SMU_1512	AMF85049.1	Phenylalanyl-tRNA synthetase subunit alpha		1	39.27	5.87	0*

SMU_804	None	FRG domain-containing protein	1		47.75	6.02	0
SMU_2091c	AMF86342.1	DNA mismatch repair protein MutS	1		95.28	5.18	0*
SMU_1043c	AMF85440.1	Phosphate acetyltransferase	1		36.35	4.92	0*
SMU_445	AMF85952.1	Glycyl-tRNA synthetase subunit alpha	1		35.06	4.93	0*
SMU_1102	AMF85388.1	Putative phospho-beta-glucosidase	1		55.1	5.13	0*
SMU_1422	AMF85120.1	Pyruvate dehydrogenase E1 component subunit beta	1		37.14	4.86	0
SMU_1623c	AMF84953.1	S1 RNA-binding domain-containing protein	1		32.21	8.99	0*
SMU_1464c	AMF85085.1	tRNA (adenine(22)-N(1))-methyltransferase		1	26	6.02	0*
SMU_791c	None	Hypothetical protein SMU_791c	1		8.7	9.46	1
SMU_1425	AMF85117.1	Chaperone protein ClpB	1		98	5.91	0*
SMU_1405c	AMF85134.1	Type II CRISPR RNA-guided endonuclease Cas9		1	156.5	8.85	0*
SMU_81	AMF86219.1	Heat shock protein GrpE	1		20.56	4.51	0*
SMU_1961c	AMF86475.1	Putative PTS system, sugar-specific	1		15.17	4.10	0

		enzyme IIA component					
SMU_444	None	Hypothetical protein SMU_444		1	3.87	9.63	0
SMU_96	AMF86209.1	Probable DNA-directed RNA polymerase subunit delta		1	22.46	3.71	0*
SMU_1651	AMF84931.1	Arsenate reductase family protein	1		13.41	5.82	0*
SMU_485	AMF85918.1	Conserved hypothetical protein	1		30.92	5.63	3
SMU_1708	AMF84878.1	Potassium transporter peripheral membrane protein	1		50.68	6.96	2
SMU_1538	AMF85024.1	Glucose-1-phosphate adenylyltransferase	1		41.9	4.72	0*
SMU_1269	AMF85237.1	Phosphoserine phosphatase SerB	1		23.68	4.84	0*
SMU_1560	AMF85005.1	EamA family transporter	1		14.71	8.66	5
SMU_780	AMF85658.1	Chorismate synthase	1		42.61	6.24	0*
SMU_1066	AMF85420.1	GMP synthase [glutamine-hydrolyzing]	1		57.2	4.88	0*
SMU_844	AMF85602.1	GNAT family N-acetyltransferase	1		20.8	9.52	0*
SMU_818	AMF85628.1	30S ribosomal protein S21	1		6.9	11.29	0*
SMU_1303c	AMF85207.1	Dipeptidase	1		53	4.97	0*
SMU_322c	AMF86056.1	Glucose-1-phosphate uridylyltransferase	1		33.8	5.42	0*

SMU_1496	AMF85057.1	Galactose-6-phosphate isomerase subunit LacA	1		15.6	6.07	0
SMU_406c	AMF86674.1	Cof-type HAD-IIB-family hydrolase	1		31.15	9.26	0
**YidC2CT**							
**Accession**		**Protein name**	**No. of Spots**		**Molecular Weight (Da)**	**Theoretical pI**	**No. of TM domains**
			**Red**	**Yellow**			
SMU_2167	AMF86415.1	50S Ribosomal Protein L2	8	2	30.2	10.64	0*
SMU_1581	AMF84989.1	DNA polymerase III, gamma/tau subunit	6	3	62.15	5.10	0*
SMU_1601	AMF84971.1	6-phospho-beta-D-glucosidase	3	4	55.37	5.17	0*
SMU_906	AMF85551.1	ABC transporter ATP-binding protein	4	3	66.82	6.44	5
SMU_1357		Putative transposase fragment	2	3	9.5	5.26	0
SMU_444		Hypothetical protein SMU_444	2	1	3.87	9.63	0
SMU_1417c	AMF85125.1	Putative oleoyl-acyl carrier protein thioesterase		2	28.72	5.70	0*
SMU_169	AMF86159.1	50S ribosomal protein L13	1	1	16.2	9.87	0*
SMU_1190	AMF85314.1	Pyruvate kinase	1	1	54.3	5.09	0*
SMU_260	AMF86110.1	Nitroreductase family protein		2	22.37	4.95	0*
SMU_651c	AMF85769.1	Putative ABC transporter, substrate-binding protein	1	1	36.7	9.17	1
SMU_486	AMF85917.1	Histidine kinase		2	38	5.80	2

SMU_15	AMF84978.1	Cell division protein FtsH	2		71.6	6.54	2
SMU_1247	AMF85264.1	Enolase		1	46.8	4.67	0*
SMU_82	AMF86218.1	Molecular chaperone DnaK		1	65.2	4.58	0*
SMU_1342	AMF85178.1	Non-ribosomal peptide synthetase		1	313.43	5.28	0*
SMU_123	AMF86196.1	DNA polymerase III PolC		1	165.46	5.11	0*
SMU_683	AMF85739.1	Putative ATP-binding protein	1		126.4	5.08	0*
SMU_2001	AMF86438.1	DNA-directed RNA polymerase subunit alpha		1	34.54	4.76	0*
SMU_358	AMF86024.1	30S ribosomal protein S7		1	17.79	10.39	0*
SMU_515	AMF85885.1	Oleate hydratase		1	67.7	6.11	0
SMU_2011	AMF86427.1	50S ribosomal protein L6		1	19.42	9.70	0*
SMU_2018	AMF86421.1	30S ribosomal protein S17		1	10.02	10.18	0*
SMU_2042	AMF86396.1	Dextranase		1	94.42	4.99	1
SMU_675	AMF85747.1	PTS system transporter protein EI	1		63.35	4.57	0*
SMU_1512	AMF85049.1	Phenylalanyl-tRNA synthetase subunit alpha		1	39.27	5.87	0*
SMU_469	AMF85931.1	Holliday junction-specific endonuclease	1		22.8	9.75	0
SMU_1464c	AMF85085.1	tRNA (adenine(22)-N(1))-methyltransferase		1	26	6.02	0*

SMU_1405c	AMF86235.1	Type II CRISPR RNA-guided endonuclease Cas9		1	156.52	8.85	0*
SMU_1599	AMF84973.1	Transcriptional regulator		1	76.69	5.89	0*
SMU_96	AMF86209.1	Probable DNA-directed RNA polymerase subunit delta		1	22.46	3.71	0*
SMU_1216c	AMF85294.1	Putative amino acid ABC transporter, permease protein		1	24.29	8.76	6
SMU_355	AMF86027.1	CMP-binding factor		1	37.07	5.45	0*
SMU_61	AMF86235.1	Transcriptional regulator	1		35.22	5.07	0*
SMU_1060	AMF85426.1	Signal recognition particle protein	1		57.03	8.80	0*
SMU_1485c	None	Putative endonuclease	1		66.35	5.06	0*
SMU_1422	AMF85120.1	Pyruvate dehydrogenase E1 component subunit beta	1		37.14	4.86	0

*Proteins identified as membrane-associated by Mishra et al. 2019 Molecular Oral Microbiology

Green spots contained GST-YidC1CT, red spots contained GST-Yid2CT, and yellow spots contained both.
