# Supplementary material for: Protein Interactomes of Streptococcus mutans YidC1 and YidC2 Membrane Protein Insertases Suggest SRP Pathway-Independent- and -Dependent Functions, Respectively
Source: mSphere. 2021 Mar 3;6(2):e01308-20. doi: 10.1128/mSphere.01308-20 (PMC8546722; doi:10.1128/mSphere.01308-20)
Supplement: TABLE S5 [file msphere.01308-20-st005.pdf]

Table S5.

Plasmids	Description	Source/Reference
Streptococcus mutans		
NG8	Wild-type (serotype c)	Knox et al., 1986
UA159	Wild-type (serotype c)	Ajdic et al., 2002
PC398	NG8 *ΔyidC2::ermB*	Laboratory stock
SP15	NG8 *ΔyidC1::aad9*	Palmer et al., 2012
AH378	NG8 *ΔyidC2::kan*	Hasona et al., 2005
AH307	NG8 *ΔftsY::kan*	Hasona et al., 2005
AH329	NG8 *Δffh::ermB*	Hasona et al., 2005
PC381	NG8 *ΔyidC1::aad9 Δffh::ermB*	Laboratory stock
Escherichia coli		
C2987	*fhuA2 Δ(argF-lacZ)U169 phoA glnV44 f80Δ(lacZ)M15 gyrA96 recA1 relA1 endA1 thi-1 hsdR17*	NEB
C2992	F' *proA*$^+$*B*$^+$ *lacI*^{q}*Δ(lacZ)M15 zzf::Tn10 (Tet*^{R}*) / fhuA2Δ(argF-lacZ)U169 phoA glnV44 80Δ(lacZ)M15 ϕgyrA96 recA1 endA1 thi-1 hsdR17*	NEB
BL21 (DE3)	F– *omp*T *hsdS*$_B$ (r_B–, m_B–) *gal dcm* (DE3)	ThermoFisher Scientific
BL21 Star™ (DE3)	F- *ompT hsdSB (rB-, mB-) gal dcm rne131* (DE3)	ThermoFisher Scientific
BTH101	F- *cya-99 araD139 galE15 galK16 rpsL1 (Str*^{R}*), hsdR2 mcrA1 mcrB1*	Euromedex, France
Plasmids		
pGEX-4T-2	GST-fusion expression vector, Amp^R	GE Healthcare Life Sciences
pET30c	Expression vector, Kan^R	Novagen
pET151D-TOPO	Expression vector, Amp^R	Invitrogen
pET151D-TOPO-*secA*	pET151-D-TOPO containing *secA*	This work
pET30c-L2	pET30c containing rplB	This work
pET30c-trL2	pET30c containing rplB	This work
pGEX-4T2-yidC1-Cterm	pGEX-4T-2 containing 682-816 bp of *yidC1*.	This work
pGEX-4T2-yidC2-Cterm	pGEX-4T-2 containing 742-933 bp of *yidC2*.	This work

pKT25	Cloning and expression vector, pSU40 derivative with T25 domain of CyaA, Kan^R	Euromedex, France.
pKNT25	Cloning and expression vector, pSU40 derivative with T25 domain of CyaA, Kan^R	Euromedex, France.
pUT18C	Cloning and expression vector, pUC19 derivative with T18 domain of Cya, Amp^R	Euromedex, France.
pUT18	Cloning and expression vector, pUC19 derivative with T18 domain of Cya, Amp^R	Euromedex, France.
pKT-Zip	pKT25 plasmid with T25 domain of Cya fused in frame with leucine zipper of GCN4, Kan^R	Euromedex, France.
pUT-Zip	pUT18C plasmid with T18 domain of Cya fused in frame with leucine zipper of GCN4, Amp^R	Euromedex, France.
pSM349	pKT25 containing T25 domain of Cya fused in frame with YidC1, Kan^R	This work
pSM352	pKT25 containing T25 domain of Cya fused in frame with YidC2, Kan^R	This work
pSM355	pKT25 containing T25 domain of Cya fused in frame with Ffh, Kan^R	This work
pSM359	pKT25 containing T25 domain of Cya fused in frame with YajC, Kan^R	This work
pSM361	pUT18C containing T18 domain of Cya fused in frame with YidC2, Amp^R	This work
pSM363	pUT18C containing T18 domain of Cya fused in frame with Ffh, Amp^R	This work
pSM366	pUT18C containing T18 domain of Cya fused in frame with YajC, Amp^R	This work
pSM377	pKT25 containing T25 domain of Cya fused in frame with SecG, Kan^R	This work
pSM383	pUT18C containing T18 domain of Cya fused in frame with SecE, Amp^R	This work
pSM386	pUT18C containing T18 domain of Cya fused in frame with Smu_286, Amp^R	This work

pSM388	pUT18C containing T18 domain of Cya fused in frame with YidC1-C termninal tail, Amp^R	This work
pSM391	pUT18C containing T18 domain of Cya fused in frame with YidC2-C termninal tail, Amp^R	This work
pSM395	pUT18C containing T18 domain of Cya fused in frame with SecG, Amp^R	This work
pSM396	pUT18C containing T18 domain of Cya fused in frame with YlxM, Amp^R	This work
pSM399	pUT18C containing T18 domain of Cya fused in frame with Smu_591c Amp^R	This work
pSM403	pKT25 containing T25 domain of Cya fused in frame with smu_1276c, Kan^R	This work
pSM405	pKT25 containing T25 domain of Cya fused in frame with FtsY, Kan^R	This work
pSM419	pUT18 containing T18 domain of Cya fused in frame with HlyX, Amp^R	This work
pSM422	pUT18 containing T18 domain of Cya fused in frame with RopA, Amp^R	This work
pSM428	pKT25 containing T25 domain of Cya fused in frame with LemA, Kan^R	This work
pSM429	pKNT25 containing T25 domain of Cya fused in frame with DnaK, Kan^R	This work
pSM432	pUT18C containing T18 domain of Cya fused in frame with ribosomal protein L2, Amp^R	This work
pSM441	pUT18C containing T18 domain of Cya fused in frame with SecY, Amp^R	This work
