# Supplementary material for: Protein Interactomes of Streptococcus mutans YidC1 and YidC2 Membrane Protein Insertases Suggest SRP Pathway-Independent- and -Dependent Functions, Respectively
Source: mSphere. 2021 Mar 3;6(2):e01308-20. doi: 10.1128/mSphere.01308-20 (PMC8546722; doi:10.1128/mSphere.01308-20)
Supplement: TABLE S6 [file msphere.01308-20-st006.pdf]

Table S6.

Name	Sequence (5'→3')	Restriction enzyme	Direction	Location
NL5F	ggaacggatcccaggtcttccagattctgttg	BamHI	Fwd	+682 to +702 bp of *yidC1*.
NL5R	ccgtagtcgacttattttctctttttatgtgctttc	SalI	Rev	+816 to +792 bp of *yidC1*.
NL6F	ggaacggatccacaaaccatatcattaaaccaaaat	BamHI	Fwd	+742 to +766 bp of *yidC2*.
NL6Rb	ccgtagtcgacttattgcttatggtgacgctgt	SalI	Rev	+933 to +912 bp of *yidC2*.
PL69F	cagaagtccatatgggtattaaagtttacaaaccaac	NdeI	Fwd	+2 to +26 bp of *rplB*.
PL69R	tggaaatactcgagtttttcgttacgacgacgaat	XhoI	Rev	+837 to +817 bp of *rplB*.
PL71R	tggaaatactcgagcttccaacggctgcgtcctgc	XhoI	Rev	+642 to +622 bp of *rplB*.
SM447	gcgggatcccAGCGATGCTTGGGAGAAGTTTG	BamHI	Fwd	+91 to +112 bp of *yidC1*.
SM448	cttaggtacccgTTTTCTCTTTTTATGTGCTTTCTTTTTAGCA	KpnI	Rev	+813 to +783 bp of *yidC1*.
SM449	gcgggatcccAAACCAACTGGTGAAGGTTG	BamHI	Fwd	+91 to +110 bp of *yidC2*.
SM450	cttaggtacccgTTGCTTATGGTGACGCTG	KpnI	Rev	+930 to +913 bp of *yidC2*.
SM451	gcgggatcccAAAGATGTACAGGAAGTCACC	BamHI	Fwd	+76 to +96 bp of *ffh*.
SM452	cttaggtacccgTTTTTTACGTTTTTTGGCTTTTTTCATTC	KpnI	Rev	+1548 to +1520 bp of *ffh*.
SM453	gcgggatcccATGTCTTATACTACAATTATCATG	BamHI	Fwd	+1 to +24 bp of *yajC*.
SM454	cttaggtacccgTTCTTCAATGGCAGATTCA	KpnI	Rev	+381 to +363 bp of *yajC*.
SM513	gcgggatcccATGAAATTCATTGGTGGCGTTTTTTC	BamHI	Fwd	+ 1 to +26 bp of *secE*.
SM514	cttaggtacccgAAAACGTTTTAATAATTCAGCCAATC	KpnI	Rev	+174 to +149 bp of *secE*.
SM515	gcgggatcccATGTACAACTTTTTAGTAACAGCATTG	BamHI	Fwd	+1 to +27 bp of *secG*.
SM516	cttaggtacccgTTTACTTGATAAAACAACAATTGCCAAG	KpnI	Rev	+234 to +207 of *secG*.
SM519	gcgggatcccATGGAGATCGAAAAAACCAATC	BamHI	Fwd	+1 to +22 bp of *ylxM*.

SM520	cttaggtacccgGTCTCTATTATCAATAGTCGTC	KpnI	Rev	+330 to +309 bp of *ylxM*.
SM521	gcgggatcccCAGGTCTTCCAGATTCTG	BamHI	Fwd	+682 to +699 bp of *yidC1*.
SM522	cttaggtacccgTTTTCTCTTTTTATGTGCTTTC	KpnI	Rev	+813 to +792 bp of *yidC1*.
SM523	gcgggatcccACAAACCATATCATTAAACCAAAATTG	BamHI	Fwd	+742 to +768 bp of *yidC2*.
SM524	cttaggtacccgTTGCTTATGGTGACGCTG	KpnI	Rev	+930 to +913 bp of yidC2.
SM525	gcgggatcccTCAACAACGGATTCTATTGAAG	BamHi	Fwd	+49 to +70 bp of *ftsY*.
SM526	cttaggtacccgTAGCAATCCTTCTAAAAGTCCTC	KpnI	Rev	+1494 to +1472 bp of *ftsY*.
SM527	gcgggatcccATGTCTAGCGGAATTGTATTG	BamHI	Fwd	+1 to +21 bp of *SMU_1276c*.
SM528	cttaggtacccgAGTAACGCCGGGTTCTAC	KpnI	Rev	+1674 to +1657 bp of *SMU_1276c*.
SM529	gcgggatcccATGAAGAAAAAAGTTATTATTACAAC	BamHI	Fwd	+1 to +26 bp of *SMU_591c*.
SM530	cttaggtacccgATAACTGTTAACATAACCTACTC	KpnI	Rev	+1062 to +1040 bp of *SMU_591c*.
SM531	gcgggatcccGTCATAGCCGTTAACATTCTC	BamHI	Fwd	+31 to +51 bp of *SMU_286*.
SM532	cttaggtacccgATTAAACAGGTTATAATAGAAACCTTG	KpnI	Rev	+2280 to +2254 bp of *SMU_286*.
SM535	gcgggatcccATGGGTAGCCAGTCCTTGATC	BamHI	Fwd	+13 to +30 bp of *SMU_1693*.
SM536	cttaggtacccgGTCTTTTTCATCTTCCTTTTCTTC	KpnI	Rev	+1335 to +1312 bp of *SMU_1693*.
SM537	gcgggatcccATGGGTATTAAAGTTTACAAAC	BamHI	Fwd	+2 to +22 bp of *rplB*.
SM538	cttaggtacccgTTTTTCGTTACGACGAC	KpnI	Rev	+837 to +821 bp of *rplB*.
SM541	gcgggatcccATGTCTACATCATTTGAAAAC	BamHI	Fwd	+1 to +21 bp of *ropA*.

SM542	cttaggtacccgTTTAACTTTAGCAGAATCAG	KpnI	Rev	+1281 to +1262 bp of *ropA*.
SM543	gcgggatcccATGTCTAAAATTATTGGTATTGATTTAG	BamHI	Fwd	+1 to +28 bp of *dnaK*.
SM544	cttaggtacccgTTTCTCCGTAAACTCTCCATC	KpnI	Rev	+1836 to +1816 bp of *dnaK*.
SM547	gcgggatcccATGTTGATTTGGATTATTTTAGTTATTG	BamHI	Fwd	+1 to +28 bp of *lemA*.
SM548	cttaggtacccgAGCCATATCTCCAAAGTC	KpnI	Rev	+558 to +541 bp of *lemA*.
SM567	atgcctgcaggATGGACGCCCTAAAGGTGAAAAGTG	PstI	Fwd	+1 to +25 bp of *secY*.
SM568	cttaggatcccgTTCTGTAACATTCATAAAACCAACATATTTTC	BamHI	Rev	+1302 to +1279 bp of *secY*.
