# Supplementary material for: Protein Interactomes of Streptococcus mutans YidC1 and YidC2 Membrane Protein Insertases Suggest SRP Pathway-Independent- and -Dependent Functions, Respectively
Source: mSphere. 2021 Mar 3;6(2):e01308-20. doi: 10.1128/mSphere.01308-20 (PMC8546722; doi:10.1128/mSphere.01308-20)
Supplement: TEXT S1 [file msphere.01308-20-s0001.pdf]

Supplemental Methods

Plasmid construction

The gene encoding 50S ribosomal protein L2 was amplified by PCR using primer PL69F and PL69R (Table S6). PCR products were digested with *Nde*I and *Xho*I and ligated to corresponding restriction enzyme sites in the pET30c vector. DNA encoding the truncated 50S ribosomal protein L2 derivative (trL2) (start-bp642) was amplified with primers PL69F and PL71R (Table S6). PCR products were subcloned into pET30c, and used to transform C2987 competent cells with selection on kanamycin. Subsequent cloning was carried out in *E. coli* strain BL21 DE3 cells (ThermoFisher Scientific) for protein expression and purification. Similarly, the gene encoding *secA* was PCR-amplified from UA159 genomic DNA and subcloned into pET151D-Topo with an N-terminal his tag and a TEV cleavage site. The resultant plasmid, pET151D-secA, was used to transform *E. coli* strain BL21 Star™ (ThermoFisher Scientific) cells for *secA* expression and protein purification.

Expression and purification of recombinant proteins. Recombinant *E. coli* were induced to produce 50S ribosomal protein L2 or trL2 with 0.05 mM IPTG at RT overnight. Recombinant *E. coli* were induced to produce SecA with 0.3 mM IPTG overnight at 16°C. Bacterial cells were harvested by centrifugation at 11,325 x *g* for 15 min, and resuspended in 25 ml 50 mM sodium phosphate, 300 mM sodium chloride,10 mM imidazole, pH 7.4, supplemented with 1 mM phenylmethylsulfonyl fluoride (PMSF) (Acros Organics) and protease inhibitor cocktail (1 mini tablet/25 ml) (Roche Diagnostics GmbH). Cell lysis was performed using an Avestin EmulsiFlex-C5 high-pressure homogenizer (Avestin Inc., Ottawa, Ontario, Canada) at a pressure of 15,000-20,000 p.s.i. for three cycles. Cell debris was removed by centrifugation and the supernatant was filtered through a 0.22 µm syringe filter (Merck Millipore). Recombinant

proteins were purified on an AKTA Purifier system (GE Healthcare) using a HiTrap TALON column and eluted with 50 mM sodium phosphate, 300 mM sodium chloride, Ph. 7.4, containing 150 mM imidazole.

Enzyme-linked immunosorbent assay (ELISA). Ninety-six-well-polystyrene plates (Costar, Corning, NY) were coated with 400 ng of purified GST-YidC1CT or GST-YidC2CT, or GST as a negative control. Wells were blocked with 2% Skim Milk (BBL, Becton Dickinson) in PBST (phosphate-buffered saline containing 0.3% Tween 20) for 2 h at 37°C. Two-fold serial dilutions of purified recombinant ribosomal protein L2, truncated L2 (trL2) or SecA, beginning at 400 ng, or 100 μl of PBST buffer only, were added to the wells and incubated for 2 h at 37°C. Unbound proteins were washed away with PBST. Overlaid or coated proteins were detected with specific polyclonal rabbit antisera (SecA, GST, GST-YidC1CT and GST-YidC2CT) or commercial anti-his mouse antisera (L2 and trL2) (Invitrogen), followed by horseradish peroxidase (HRP)-labeled goat anti rabbit IgG or anti mouse IgG and development with *o*-phenylenediamine. Adequate coating of wells with GST-YidC1CT or GST-YidC2CT was established using polyclonal rabbit antisera against the YidC1 and YidC2 CTs as the primary antibody.
